# Antibiotic Use in Children with Acute Respiratory or Ear Infections: Prospective Observational Comparison of Anthroposophic and Conventional Treatment under Routine Primary Care Conditions

**DOI:** 10.1155/2014/243801

**Published:** 2014-11-18

**Authors:** Harald J. Hamre, Anja Glockmann, Reinhard Schwarz, David S. Riley, Erik W. Baars, Helmut Kiene, Gunver S. Kienle

**Affiliations:** ^1^Institute for Applied Epistemology and Medical Methodology, University of Witten-Herdecke, Zechenweg 6, 79111 Freiburg, Germany; ^2^Paediatric Practice, Quellengasse 42, 8010 Graz, Austria; ^3^Integrative Medicine Institute, Overton Street, Portland, OR 97210, USA; ^4^University of Applied Sciences Leiden, Zernikedreef 11, 2333 CK Leiden, The Netherlands; ^5^Louis Bolk Instituut, Hoofdstraat 24, 3972 LA Driebergen-Rijsenburg, The Netherlands

## Abstract

Children with acute respiratory or ear infections (RTI/OM) are often unnecessarily prescribed antibiotics. Antibiotic resistance is a major public health problem and antibiotic prescription for RTI/OM should be reduced. Anthroposophic treatment of RTI/OM includes anthroposophic medications, nonmedication therapy and if necessary also antibiotics. This secondary analysis from an observational study comprised 529 children <18 years from Europe (AT, DE, NL, and UK) or USA, whose caregivers had chosen to consult physicians offering anthroposophic (A-) or conventional (C-) treatment for RTI/OM. During the 28-day follow-up antibiotics were prescribed to 5.5% of A-patients and 25.6% of C-patients (*P* < 0.001); unadjusted odds ratio for nonprescription in A- versus C-patients 6.58 (95%-CI 3.45–12.56); after adjustment for demographics and morbidity 6.33 (3.17–12.64). Antibiotic prescription rates in recent observational studies with similar patients in similar settings, ranged from 31.0% to 84.1%. Compared to C-patients, A-patients also had much lower use of analgesics, somewhat quicker symptom resolution, and higher caregiver satisfaction. Adverse drug reactions were infrequent (2.3% in both groups) and not serious. Limitation was that results apply to children of caregivers who consult A-physicians. One cannot infer to what extent antibiotics might be avoided in children who usually receive C-treatment, if they were offered A-treatment.

## 1. Background

### 1.1. Acute Respiratory Tract Infections and Otitis Media (RTI/OM)

Acute respiratory tract infections and otitis media (RTI/OM) are frequent among children [1] and are commonly treated with antibiotics [2–8]. In randomised trials, antibiotics have only small or negligible short-term effects on OM and RTI such as pharyngitis, bronchitis, laryngitis, and common cold, comparable to their side-effect potential [9–12]. Antibiotic treatment as secondary prophylaxis in order to prevent complications of RTI/OM is difficult to justify in developed countries, where these complications are rare [13, 14]. Furthermore, antibiotic use increases antimicrobial resistance [15], increases the recurrence rate of OM [16], and may be a risk factor for paediatric asthma [17–20], atopic eczema [21], and inflammatory bowel disease [22–24]. The European Commission has recently proposed an action plan against the rising threats from antimicrobial resistance, which is estimated to cause 25,000 human deaths as well as extra healthcare costs and productivity losses of at least 1.5 billion Euro annually [25].

Because of these concerns, reduction of antibiotic prescription for RTI/OM has long been advocated [26–30]. Strategies to reduce antibiotic use include educational interventions towards physicians and patients [31], rapid antigen testing to identify viral disease [32], and delayed antibiotic prescription [33, 34]. In addition, using analgesics instead of antibiotics has been recommended [35], but analgesics may also pose risks [36–41].

### 1.2. Complementary Treatment for RTI/OM, Background for this Study Analysis

Some physicians prescribe complementary treatment for children with RTI/OM, such as herbal [42–44], homeopathic [45–47], or anthroposophic [48–51] medicinal products. Complementary treatment might reduce the need for antibiotics, but antibiotic reduction should not be accompanied with delayed short term recovery or increased complication rates [52]. We have previously investigated these issues in a prospective observational study in primary care, showing low antibiotic use with at least comparable short-term resolution in adults and children treated for RTI/OM by physicians offering treatment with anthroposophic medicine (AM, see Section 1.3), compared to physicians offering conventional treatment [53]. However, in the primary analysis adults and children were assessed together [53]. There is a specific need for data on medication use and safety in children [54] and the problem of inappropriate use of antibiotics for RTI/OM is particularly pertinent in children, for several reasons (high prevalence of RTI/OM and associated antibiotic prescription, lack of detailed data on antibiotic use and resistance, and special issues in children such as parental or physician anxiety for complications and parental stress from attendance to the sick child [55]). On the other hand, studies with children can be difficult to conduct, again because of special issues with children such as ethical concerns [56].

We therefore performed a secondary analysis of the study subgroup of children with a special focus on antibiotic use. Since antibiotic use for RTI/OM in children has to some extent diminished after the data in our study were collected in 1999-2000 [2–8, 57], we also compared antibiotic use in our study analysis descriptively to antibiotic use in recent observational studies of children with RTI/OM.

### 1.3. Anthroposophic Medicine and Childhood Infections

AM is a physician-provided integrative multimodal therapy system founded by Steiner and Wegman [58]. AM is based on the cognitive methods and cognitive results of anthroposophy [59]. According to the anthroposophic understanding of man and nature, four different classes of formative forces can be discerned as follows: (1) in minerals, formative forces of physicochemical matter; (2) in plants, formative vegetative forces interact with material forces, bringing about and maintaining the living form; (3) in animals with sensory and motor systems and with a corresponding inner life, a further class of formative forces (anima, soul) interact with material and vegetative forces; (4) in the human organism with its individual mind and capacity of thinking, another class of formative forces (Geist, spirit) interact with the material, vegetative, and animal forces. The interactions of these forces are understood to vary between different regions and organs in the human body, resulting in a complex equilibrium [59]. This equilibrium can be distorted in various forms of human disease. Acute RTI/OM and other childhood infections are seen as part of a developmental process, involving the working of the spiritual forces on the material and vegetative forces. Accordingly, fever is not routinely suppressed with analgesics, and antibiotics are only prescribed if strongly needed. For alleviation of fever and other symptoms, priority is given to AM medications and nonmedication treatment such as steam inhalations, nasal lavage, and various external applications (cold dressings on legs to lower temperature, local compresses, etc.) [60–64].

AM medications are prepared from plants, minerals, animals, and from chemically defined substances according to Good Manufacturing Practice and national drug regulations; quality standards of raw materials and manufacturing methods are described in the Anthroposophic Pharmaceutical Codex [65]. Toxicologically relevant starting materials (e.g., aconite, cinnabar) are highly diluted according to safety requirements of European regulations [66]. The available evidence suggests that AM medications are generally well tolerated, with infrequent adverse reactions of mostly mild to moderate severity [67, 68].

AM treatment for RTI/OM can be standardised (e.g., one AM medication for a given indication) or individualised (tailored to individual needs, involving one or several AM medications and/or nonmedication AM therapies) [62–64].

## 2. Methods

### 2.1. Design, Objective, and Research Questions

This is a subgroup analysis of data from a prospective observational comparative study in primary care (IIPCOS-Anthroposophy, International Integrative Primary Care Outcomes Study) [53]. The IIPCOS-Anthroposophy study comprised adults and children treated for acute RTI/OM under routine primary care conditions. Two patient groups were compared, according to the treatment offered by the physicians whom the patients or caregivers consulted: physicians offering AM therapy (A-physicians) and physicians offering conventional therapy (C-physicians). A descriptive, unadjusted analysis of clinical outcomes in adults and children was preplanned and has been summarised in the primary publication [53], while the present detailed analysis of the children, with a special focus on antibiotic use, was secondary.

The objective of this analysis was to compare antibiotic use in children treated for RTI/OM under routine primary care conditions by A-physicians or C-physicians, respectively, and to investigate factors associated with antibiotic use. The analysis addressed the following research questions.

- Among children treated for RTI/OM under routine primary care conditions, was treatment by A-physicians associated with lower antibiotic or analgesic use, compared to treatment by C-physicians?
- To what extent could differences in antibiotic or analgesic use be explained by demographic differences, differences in baseline morbidity, previous treatment by the physician, caregiver confidence in the physician's professional skills at baseline, or consultation length?
- Was treatment by A-physicians associated with safety problems (adverse drug reactions, complications from RTI/OM, or delayed short-term recovery from RTI/OM)?
- How does antibiotic use in this analysis of the IIPCOS-Anthroposophy study, with data collected 1999-2000, compare to antibiotic use in recent observational studies in similar settings with similar patients?

### 2.2. Setting, Participating Physicians, Patients, and Treatment

The IIPCOS-Anthroposophy study was conducted 1999-2000 in primary care practices in Austria, Germany, The Netherlands, UK, and the United States. A-physicians (prescribing AM medications to at least 75% of patients with acute RTI/OM) were recruited through national AM physicians' associations; C-physicians (not prescribing AM medications) were recruited by HomInt research network. Patients were treated according to the physicians' discretion.

Inclusion criteria for this analysis were (1) age ≥1 month to 17 years (in The Netherlands the lower age limit was 4 years as requested by the local ethics committee), (2) chief complaint of sore throat, cough, or ear pain, and (3) onset of chief complaint within 7 days. (The primary analysis, comprising adults and children, had also included patients with a chief complaint of runny nose and sinus pain [53]. These two chief complaint groups were not included in the present analysis, because each of them had <5 patients seeing conventional physicians).

Treatment for RTI/OM was evaluated as a whole system, including physician-caregiver-child interactions [53, 69] and was defined as the consultation with the A- or C-physician at baseline and any medication or nonmedication treatment prescribed for RTI/OM at baseline or during a 28-day follow-up period.

### 2.3. Main Outcomes

Primary outcome was prescription of antibiotics (Anatomical Therapeutic Chemical [ATC] Index J01 antibacterials for systemic use) on days 0–28. Antibiotic prescription rates in this study analysis were also descriptively compared to antibiotic prescription rates in recent observational studies of children with acute RTI/OM in primary care (see the subsection “Comparison to Antibiotic Prescription in Other Studies”).

The other main outcomes were

- prescription of analgesics (ATC-Index N02 Analgesics) on days 0–28,
- improvement within 24 hours and 3 days,
- response at days 7 and 14, a response being defined as treatment outcome = complete recovery or major improvement (response categories were complete recovery/major improvement/slight to moderate improvement/no change/deterioration),
- complete recovery at days 7 and 14,
- caregiver very satisfied with the treatment (very satisfied/satisfied/neutral/dissatisfied/very dissatisfied) at all follow-ups,
- caregiver would choose the same therapy again for the health problem of the child (yes/no) at all follow-ups.

### 2.4. Further Outcomes

Further outcomes were prescription of other medications, compliance with medication prescription, response, and recovery at day 28, caregiver satisfaction with the physician, adverse drug reactions, and serious adverse events.

### 2.5. Data Collection

On day 0, physicians documented chief complaint (name, duration, previous episodes within last year, diagnosis, severity: 0 = not present, and 4 = very severe), severity of four complaint-related symptoms (cough: five symptoms), concomitant diseases, caregivers willingness to their child being randomised, and therapies. On days 7, 14, and 28, caregivers were interviewed by telephone about treatment outcome, time to first improvement (number of hours or days), medication use and safety, and caregiver satisfaction. The interviewers were not blinded towards the treatment setting (AM or conventional); caregivers were informed about the planned comparison of treatment regimens. The caregivers' responses were not made available to the physicians.

### 2.6. Data Analysis

#### 2.6.1. Comparison of Anthroposophic and Conventional Groups in This Study

Patients fulfilling all eligibility criteria with at least one follow-up interview were included in the analysis. Data analysis was performed using IBM SPSS Statistics 19 (International Business Machines Corp., Armonk, NY, USA) and StatXact 9.0.0 (Cytel Software Corporation, Cambridge, MA, USA).

For patients with complete recovery on days 7 or 14, study participation was terminated and last observations were carried forward for analysis of subsequent follow-ups. Follow-up data missing for other reasons were also replaced by last observation carried forward, when available. Otherwise, missing data were not replaced.

Bivariate analyses of independent samples were performed using Fisher's exact test for dichotomous data, Fisher-Freeman-Halton test for multinomial data, and *t*-test for continuous data, unless otherwise stated. All tests were two-tailed.

Main outcomes were analysed in subgroups pertaining to seven prognostic variables identified by systematic literature search: gender, age (<2 years, 2–5, 6–17), chief complaint, duration of chief complaint (0-1 day, >1-2, >2–7), previous episode of chief complaint within last year (yes/no), baseline symptom score (mean severity of chief complaint and complaint-related symptoms, respectively: 0–<1, 1–4), concomitant disease present at baseline (yes/no). These subgroup analyses differed from the planned analyses in the following aspects: two age subgroups (6–11, 12–17 years) and three Symptom Score severity subgroups (1-2, 2-3, 3-4) were grouped together because of low sample size.

Main outcomes were subject to multiple logistic regression analysis to adjust for all seven prognostic variables. The possibility of clustered patient sampling on the levels of individual physicians or physician practices was investigated by calculating intraclass correlation coefficients (ICC type 1,1 according to Shrout and Fleiss [70]) between the main outcomes and physicians respective physician practices. There was no evidence of clustering: (ICC ≤ 0.00, *P* = 1.000 for all analyses). A multilevel analysis, including physician or physician office level in addition to patient level, was therefore not considered necessary. The regression analyses were performed using the Binominal Logistic function; variables were included using the Enter procedure. Model assumptions were checked and verified [71, 72]. The final regression analyses differed from the planned analyses in one aspect: country was not included as a prognostic variable because three of the five countries had less than ten C-patients (Table 1).

#### 2.6.2. Descriptive Comparison to Antibiotic Use in Other Studies

Antibiotic prescription rates in this study analysis were also descriptively compared to antibiotic prescription rates in recent observational studies of children with RTI/OM. These studies were identified by systematic literature searches in Pubmed and Google Scholar (search strategies in Table 2); in addition the library of the European Surveillance of Antimicrobial Consumption Network (http://www.ecdc.europa.eu/en/activities/surveillance/ESAC-Net/Pages/index.aspx) was hand searched. Eligible for comparison were the following studies:

- from the same five countries as in this study (AT, DE, NL, UK, US),
- published in the period from 1 January 2006 to 31 December 2012,
- published in English or German language,
- reporting on children aged 0–17 years,
- in primary care settings,
- treated for acute chief complaints or diagnoses similar to this study (Table 3),
- for which antibiotic prescription rates were reported or could be calculated.

Antibiotic prescription rates in the comparison studies were defined as the number of children (or cases in studies reporting several RTI/OM episodes in each child) with antibiotic prescription for one of the diagnosis groups 1–4 (Table 3)/number of children (or cases) treated for the respective diagnosis group. If prescription rates for more than one time period were reported, only the last period was included. Prescription rates referred to days 0–28 in this study and to any time frame between 0 and 28 days from the first visit in the comparison groups.

For each of the diagnosis groups 1–4 in Table 3, the prescription rates in the A- and C-patients of this study were compared to the prescription rates in the comparison groups. The comparisons were descriptive without statistical hypothesis testing: prescription rates were not pooled or adjusted but ordered in increasing magnitude [73].

### 2.7. Quality Assurance and Adherence to Regulations

The IIPCOS-Anthroposophy study was approved by The Freiburg Ethics Commission International and by local ethics committees. The study was conducted according to the Helsinki Declaration, the International Conference on Harmonisation Good Clinical Practice guidelines, and legal requirements. Written informed consent was obtained from the legal guardians of all patients before study enrolment. This report followed the STROBE guidelines for reporting of observational studies [74].

## 3. Results

### 3.1. Participating Physicians and Patients

32 physicians (24 anthroposophic “A-physicians” + 8 conventional “C-physicians”) enrolled patients into the analysed sample, these physicians were located in Austria (*n* = 2 + 2), Germany (*n* = 6 + 2), NL (*n* = 6 + 2), UK (*n* = 2 + 2), and USA (*n* = 8 + 0) in 25 different practices in 20 different municipalities. A total of 79% (*n* = 19/24) of A-physicians and 88% (*n* = 7/8) of C-physicians were men. Physicians' qualifications were: general practitioners (17 A-physicians + 6 C-physicians), internists (2 + 2), and paediatricians (4 + 0).

### 3.2. Patient Enrolment and Follow-Up

A total of 596 children with a chief complaint of sore throat, cough, or ear pain were enrolled. 67 children were excluded from the analysis because of protocol violations (*n* = 43, one telephone interviewer had not performed the telephone follow-up interviews according to protocol) or because they had no evaluable follow-up data (*n* = 24), 529 children were evaluable (443 A-patients and 88 C-patients; a flow chart of enrolment, exclusions and follow-up interviews is presented in Supplementary Figure 1 in Supplementary Material available online at http://dx.doi.org/10.1155/2014/243801). The 529 children were enrolled by general practitioners (149 A-patients + 82 C-patients), internists (7 + 4), and paediatricians (287 + 0) (Fisher-Freeman-Halton test: *P* < 0.001). The number of children enrolled per physicians was median 5.5 (interquartile range 2.3–17.8) in the A-group and 5.0 (1.5–15.5) in the C-group (Mann-Whitney* U*-test: *P* = 0.540).

Further data on patient screening and follow-up are presented in the primary analysis of the IIPCOS-Anthroposophy study (comprising 1016 adults and children with one of five chief complaints, in contrast to this subgroup analysis comprising 529 children with one of three chief complaints) [53]. The primary analysis indicated that enrolled A-patients were representative for all eligible A-patients (while no screening data for the C-group were available) and that neither attrition bias as such, nor alternative ways of analysing missing data would change overall study results [53].

### 3.3. Baseline Characteristics

#### 3.3.1. Demographics

The A- and C-groups did not differ significantly regarding gender, age, race, body mass index, household size or income, or previous treatment by study physician. The groups differed significantly regarding country (Table 1).

#### 3.3.2. Disease Status at Baseline

The A- and C-groups did not differ significantly regarding the frequency of chief complaints sore throat (*P* = 0.251) or cough (*P* = 0.196) nor the diagnoses otitis media (*P* = 0.144) or pharyngitis/tonsillitis (*P* = 0.063). Furthermore, the groups did not differ regarding the presence of fever ≥38.5°C, a chief complaint episode within last year, the presence of any concomitant disease or the ongoing use of any medication. However, a concomitant respiratory disorder and the use of asthmatics were more frequent in the C-group. No patient was using corticosteroids or antibiotics.

The A-group had significantly lower frequency of ear pain as chief complaint (*P* = 0.016) and a diagnosis of common cold/upper RTI, and a higher frequency of a diagnosis of laryngitis/tracheitis/bronchitis. Also, the A-group had shorter complaint duration, higher baseline symptom score, and more frequently a chief complaint of severe or very severe intensity (among all patients as well as in the subgroup of patients with a chief complaint of ear pain) (Table 4).

### 3.4. Attitudes, Expectations, Diagnostic Procedures, and Consultation Time

The caregivers' confidence in physician's professional skills was significantly higher in the A-group, but caregivers' confidence that the treatment would solve the medical problem was similarly high in both groups (Table 4). The caregivers were not willing that their children should be randomised if the treatment would be part of a clinical trial in 98.2% (*n* = 435/443) of A-patients and in 80.2% (*n* = 69/86) of C-patients (*P* < 0.001). The most frequent reason for refusing randomisation was a treatment preference (96.1%, *n* = 418 of 443 A-patients; 87.0%, *n* = 60 of 69 C-patients). The physicians' confidence in their diagnosis was similar in both groups, but A-physicians were more likely to base diagnosis on clinical examination than C-physicians. In more than 90% of patients of both groups the caregiver had been free to choose the physician. Consultation time was significantly longer in the A-group (Table 4).

### 3.5. Antibiotic Use

#### 3.5.1. Comparison of Anthroposophic and Conventional Groups in This Study

Antibiotics were prescribed to 17.4% of C-patients and 0.5% of A-patients on day 0 (*P* < 0.001), and to 25.6% and 5.0%, respectively, throughout days 0–28 (*P* < 0.001). In the A-group, no significant difference was found between antibiotic prescription rates in patients treated by general practitioners (4.0%, *n* = 6/149), internists (14.3%, *n* = 1/7), and pediatricians (5.2%, *n* = 15/287), respectively (Kruskal-Wallis test *P* = 0.448). Further subgroup analyses and adjusted analyses of antibiotic prescription are presented in the section “Main Outcomes,” below.

#### 3.5.2. Descriptive Comparison to Antibiotic Use in Other Studies

**Figure 1:**
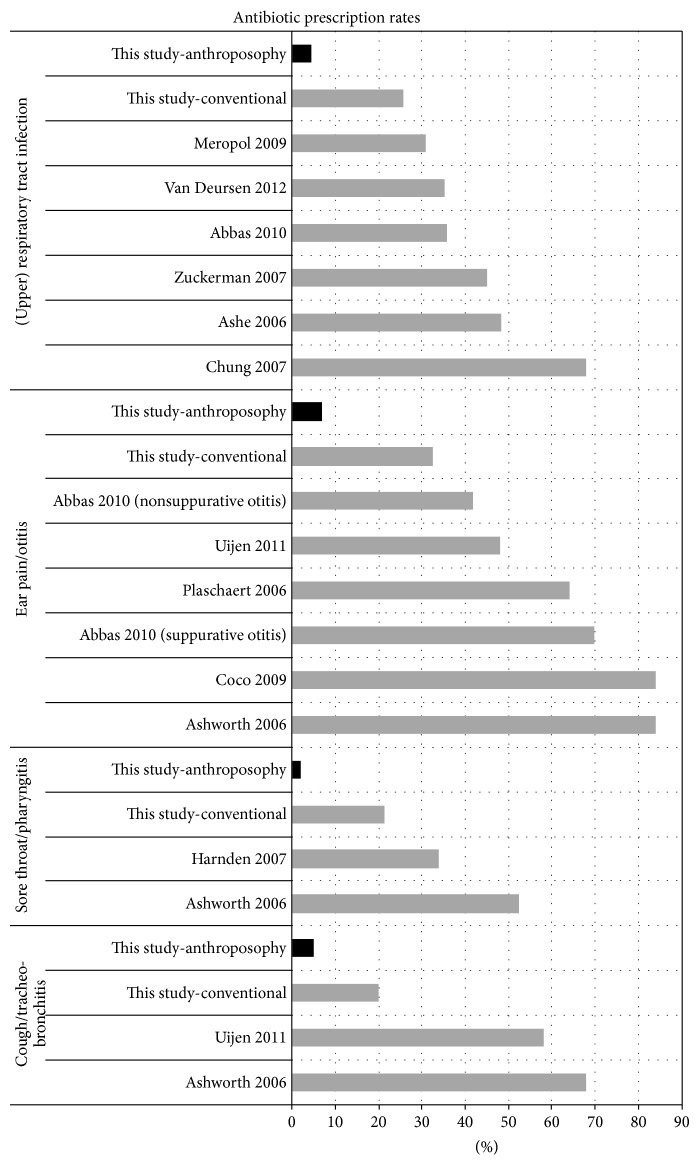
Comparison to other studies. Antibiotic prescription rates. Percentage of patients (or cases) with acute respiratory infections or otitis who were prescribed antibiotics.

Antibiotic prescription rates in this study were also compared to antibiotic prescription rates in recent observational studies of children with acute RTI/OM (see Methods for details). The literature searches identified 36 potentially eligible studies published 2006–2012. Of these, 25 studies did not fulfil the eligibility criteria, for the following reasons: no data on children (*n* = 7 studies), other country than in this study (*n* = 12), no data on diagnoses of this study (*n* = 4), no data on antibiotic prescription rates (*n* = 2). Eleven studies [2, 7, 8, 75–82] with a total of 16 diagnosis groups (cough/tracheitis/bronchitis: *n* = 2, sore throat/pharyngitis: *n* = 2, ear pain/otitis: *n* = 6, Upper RTI/RTI: *n* = 6) were eligible for comparisons (further data in Table 5). All 16 comparison groups had higher antibiotic prescription rates than the corresponding C-, and A-groups of this study (Figure 1). Antibiotic prescription rates ranged from 31.0% (nonspecific RTI [7]) to 84.1% (OM [77]). Compared to the rates in the A-groups, prescription rates in the comparison groups were 6.7 [80] to 13.3 [77] times higher for cough/bronchitis, 26.2 [77] to 29.2 [81] times higher for sore throat/pharyngitis, 6.1 [75] to 12.2 [77, 79] times higher for ear pain/OM, and 6.9 [7] to 15.1 [78] times higher for upper RTI/RTI.

### 3.6. Other Therapies

#### 3.6.1. Analgesics

On days 0–28, analgesics were prescribed to 25.6% (*n* = 22/86) of C-patients and 5.0% (*n* = 22/443) of A-patients (see also Supplementary Table 1). Analgesics prescribed on days 0–28 were paracetamol (11 A-patients + 22 C-patients) and choline salicylate (3 + 0). Odds ratio (A- versus C-group) for nonprescription of analgesics on days 0–28 after adjustment for seven prognostic variables (see Methods) was 12.11 (95%-CI 5.50–26.69). In an alternative analysis, the prognostic variable “fever ≥ 38.5°C or (ear pain group only) severe or very severe ear pain at baseline” was substituted for “symptom score at baseline”; adjusted OR for nonprescription of analgesics was 12.75 (95%-CI 5.79–28.12).

In the A-group, no significant difference was found between analgesic prescription rates in patients treated by general practitioners (2.7%, *n* = 4/149), internists (0.0%, *n* = 0/7), and pediatricians (3.5%, *n* = 10/287), respectively (Kruskal-Wallis test *P* = 0.804).

#### 3.6.2. Anthroposophic Medications, External Applications

AM medications were prescribed to all A-patients and no C-patient. A total of 207 different AM medications were prescribed throughout the study; the most frequent AM medications were Plantago Bronchial Balm (prescribed to *n* = 101/443 A-patients, 22.8%), Erysidoron 1 Liquid (20.1%), Cinnabar comp. Powder (17.8%), Pine Reviving Bath Milk (11.3%), Sticta Liquid (10.6%), and Aconitum comp. Eardrops (9.7%). External applications were prescribed to 30.9% (*n* = 137/443) of A-patients; this item was not documented in C-patients.

#### 3.6.3. Other Medications

Compared to the A-group, Anti-inflammatory agents and antihistamines were also prescribed significantly more often in the C-group, while cough and cold preparations were more frequently prescribed in the A-group (Supplementary Table 1).

#### 3.6.4. Duration of Prescribed Use, Confidence in and Compliance with Prescription

The medication was prescribed to be taken for an average of 11.4 days ± 6.2 in the A-group and 4.0 days ± 2.2 in the C-group (*P* < 0.001, mean difference: 7.5 days; 95%-CI: 6.7–8.2). The physicians' confidence in their prescription (range 0–10) was mean 9.2 ± 1.0 and 7.6 ± 1.7 points in A- and C-groups, respectively (*P* < 0.001, mean difference: 1.6 points, 95%-CI: 1.3–2.0). The caregivers reported being compliant with medication prescriptions throughout follow-up in 91.6% (*n* = 406/443) of A-patients and 82.6% (*n* = 71/86) of C-patients (*P* = 0.002).

### 3.7. Main Outcomes

#### 3.7.1. Main Analyses

**Figure 2:**
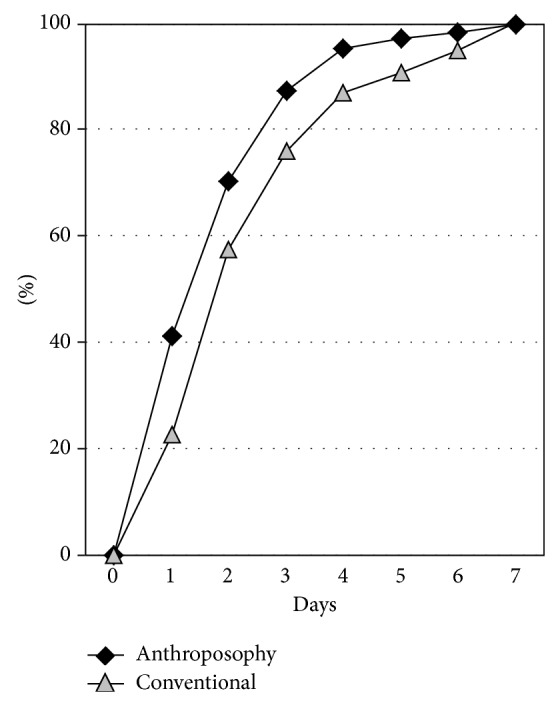
Time to first improvement. Cumulative percentage of patients with available data. Anthroposophy group: *n* = 410 and conventional group: *n* = 75.

In the unadjusted analyses of the ten main outcomes, A-patients had more favourable outcomes (lower use of antibiotics and analgesics; higher proportions of patients with first improvement within 1 and 3 days, respectively; higher proportions with major improvement and total recovery within 7 and 14 days, respectively, and higher caregiver satisfaction as well as a higher proportion of caregivers choosing the same therapy again). These differences were significant for all outcomes except recovery on day 7 (Table 6, Figure 2, Supplementary Table 2).

Unadjusted OR (A- versus C-) for the ten main outcomes was analysed in 17 subgroups pertaining to seven prognostic variables (details in Methods section, altogether 170 comparisons). These OR favoured A-patients for 166 comparisons and C-patients for 4 comparisons. The four comparisons favouring C-groups pertained to 4 different subgroups and 2 different outcomes, respectively (improvement within 3 days: one subgroup; recovery on day 7: three subgroups). In A- and C-groups alike, children with a chief complaint of sore throat or ear pain had more favourable clinical outcomes than children with a chief complaint of cough. Also, children in the age group 2–5 years had more favourable clinical outcomes than younger as well as older children (Supplementary Table 3).

The ten main outcomes were adjusted for the seven prognostic variables using multiple logistic regression analysis (see Methods for details, *n* = 527 patients with available data for all variables). The most consistent relationship between independent and dependent variables was observed for the variables “duration of chief complaint” and “chief complaint”. A longer duration of chief complaint was associated with a worse outcome in nine of the ten models; this association was significant in seven models. A chief complaint of cough was associated with a worse outcome in eight models (significant in five models). Three variables (age, previous episode of chief complaint, baseline symptom score) were significant regressors in two models each. Two variables (gender, concomitant disease at baseline) were not significant regressors in any model. Re-analysis without these two variables yielded results very similar to the main analysis. All seven variables were therefore included in the final models. As in the unadjusted analyses, all adjusted OR favoured the A-group, with significant differences for all outcomes except recovery on day 7. Compared to the unadjusted OR, the adjusted OR were very similar (<10% increase or decrease) in seven analyses, they were increased by at least 10% in two analyses, and were decreased by at least 10% in one analysis (Table 6).

#### 3.7.2. Sensitivity Analyses

In a sensitivity analysis (SA [a], Supplementary Table 4), unadjusted and adjusted OR for main outcomes were calculated after restriction of the analysed sample to patients from Austria, Germany, The Netherlands, and the UK, since no C-patients were enrolled in the USA. Results were very similar to results of the main analyses (<10% change of OR in eight analyses, OR increased by at least 10% in two analyses).

In further sensitivity regression analyses, one independent variable was substituted for another variable: baseline severity of chief complaint was substituted for baseline symptom score (SA [b], Supplementary Table 5), the number of previous episodes of chief complaint was substituted for the dichotomized variable (previous episodes: yes/no) (SA [c], Supplementary Table 5), and a concomitant respiratory disorder was substituted for any concomitant disorder (SA [d], Supplementary Table 5). SA [b] and SA [c] yielded results very similar to the main analyses (<10% change in 19 of 20 analyses), while in SA [d], OR were increased by at least 10% in four analyses and showed <10% change in six analyses.

In each of four sensitivity analyses (SA [e–h]), one additional independent variable of potential interest was included in the model: previous treatment by the study physician (SA [e], Supplementary Table 6), body mass index (SA [f], Supplementary Table 7), household size (SA [g], Supplementary Table 8), and household income (SA [h], Supplementary Table 9). These four variables were included separately in the regression models for the ten main outcomes, yielding a total of 40 analyses. In 39 analyses the additional variable was not a significant regressor, while household size was a significant regressor for caregiver satisfaction with treatment. Compared to unadjusted OR, adjusted OR were very similar (<10% change) in 23 analyses, they were increased by at least 10% in 13 analyses, and were reduced by at least 10% in 4 analyses. Of the 40 analyses, 39 OR favoured the A-group, while OR for complete recovery on day 7 after adjustment for household income (SA [h] with *n* = 253 patients with available data for households income) favoured the C-group; results were significant for antibiotic and analgesic use, for markers of early improvement (improvement after 1 and 3 days, response on day 7), for therapy satisfaction, and for caregiver choosing this therapy again in all analyses (28 OR); in addition, they were significant for response on day 14 in two out of four analyses.

All prognostic variables described so far were deemed to be unrelated to the AM therapy system. In further sensitivity analyses two additional variables which could be related to the AM therapy system were included: caregiver's confidence in the physician's professional skill and consultation length (SA [i–k], Supplementary Table 10). Confidence in the physician's professional skill was a significant regressor for six major outcomes (not for antibiotic or analgesic prescription and not for recovery on days 7 and 14) (SA [i]). Consultation length was a significant regressor for one major outcome (caregiver satisfaction with treatment) (SA [j]).

Compared to the unadjusted OR for this sample (*n* = 436 patients with available data for all variables), adjustment for the seven variables of the main models plus consultation length (SA [i]) or caregiver's confidence in physician's professional skill (SA [j]) or both (SA [k]) increased the OR for no antibiotic and no analgesic prescription in all 6 analyses (increase of at least 10% in 5 analyses); reduced the OR for five outcomes (recovery on day 7, response on days 7 and 14, caregiver's therapy satisfaction, caregiver choosing the same therapy again) by at least 10% in all 15 analyses, while effects on improvement after 1 and 3 days and response on day 7 were very small (<10% change of OR in eight of nine analyses). In these analyses (SA [i–k]) all the 30 OR favoured the A-group; results were significant for 21 OR.

### 3.8. Other Outcomes

Response rates on day 28 were similar in both groups: A-patients: 96.2% (*n* = 426/443) versus C-patients 93.0% (80/86) (*P* = 0.241), while complete recovery at day 28 was significantly more frequent in A-patients (87.6%, *n* = 388/443) than in C-patients (77.9%, *n* = 67/86), OR for recovery (A versus C) = 2.00, 95%-CI 1.12–3.58, *P* = 0.026.

Caregiver satisfaction with the physician was significantly higher in the A-group, with the proportion of caregivers being “very satisfied” at all follow-ups in the A-group 73.4% (*n* = 325/443), in the C-group 40.7% (*n* = 35/86), OR (A versus C) = 4.01, 95%-CI 2.49–6.48, *P* < 0.001. The proportion of patients whose caregivers would choose the same physician again at all follow-ups was also significantly higher in the A-group (98.9%, *n* = 438/443) than in the C-group (93.0%, *n* = 80/86), OR (A versus C) = 6.57, 95%-CI 1.96–22.04, *P* = 0.004.

Reported adverse drug reactions (adverse events with a probable or possible relationship to any medication, according to caregivers' responses) occurred in 2.3% (*n* = 10/443) of A-patients and 2.3% (*n* = 2/86) of C-patients (*P* = 1.000). The intensity of these reactions was mild in all cases but one (appetite decreased with severe intensity in one C-patient).

All reported adverse drug reactions in the A-group (*n* = 10 patients) were subject to a detailed safety analysis [83]. For three patients the reported reactions were medically confirmed (1: diarrhoea from ivy leaf extract; 2: eyelid oedema from sodium cromoglycate and/or salbutamol; 3: injection site reaction from Prunus spinosa 5% injections); all three reactions were of mild intensity and subsided within 1–3 days following withdrawal (patients 1-2) or dose reduction (patient 3) of the medication. For seven patients the reported reactions were not confirmed; in all cases the most probable cause was the primary illness or an intercurrent illness [83].

Serious Adverse Events (SAE) occurred in 0.5% (*n* = 2/443) of A-patients and 1.2% (*n* = 1/86) of C-patients (*P* = 0.413). All SAE were acute hospitalisations. SAE in A-patients: (1) asthma, mesenteric adenitis, (2) suspected meningitis (suspicion not confirmed); SAE in C-patient: tonsillectomy. At the last follow-up, all SAE had subsided. None of these SAE was related to any medication.

## 4. Discussion

### 4.1. Overall Study Findings

This was a secondary analysis of antibiotic prescription in children from a prospective observational study of primary care patients with acute RTI/OM, treated by physicians offering AM therapy (A-physicians) or conventional therapy (C-physicians) under routine clinical conditions. Treatment by A-physicians was associated with much lower use of antibiotics (prescribed to 5% versus 26% of A- and C-patients, respectively, during the four-week follow-up) and also analgesics/antipyretics (3% versus 26%). Although data had been collected in 1999-2000 and antibiotic prescription for RTI/OM has reportedly been reduced since then, antibiotic prescription rates in this study (overall 8%, in A-patients 5%) were still much lower than in similar observational studies of RTI/OM from 2006–2012 (range 31% to 84%). There was no safety problem associated with either of the two treatment regimens, and AM therapy was not associated with delayed recovery in any subgroup. On the contrary, A-patients had somewhat quicker improvement and recovery as well as higher caregiver satisfaction.

All these differences (antibiotic and analgesic prescription, improvement, recovery, caregiver satisfaction) remained after adjustment for age, gender, chief complaint, and four markers of baseline severity. Furthermore, the differences could not be explained by A-caregivers being more confident in the A-physicians' professional skills or by A-physicians having longer consultations than C-physicians.

### 4.2. Strengths and Limitations

Strengths of this study and the present analysis include a detailed assessment of baseline status; the inclusion of patients from a range of therapy settings (25 practices, 20 municipalities); the documentation of routine diagnostics and treatment in primary care settings without experimental constraints; and the widespread use of sensitivity analyses. Whereas in many other antibiotic prescription studies the available data are limited to demographics, diagnoses, and prescriptions, a strength of this study is the prospective documentation of treatment and outcomes during a follow-up period of four weeks, which is long for acute RTI/OM. This documentation enabled the assessment also of safety (adverse reactions, complications of RTI/OM, possible delayed short-term resolution due to possible under-prescription of necessary antibiotics in A-patients), which is an important finding, since AM therapy for RTI/OM has not been studied extensively.

Notably, this was not an experimental investigation of specific intervention effects; the research questions concerned behaviour (antibiotic prescription) in two different settings under naturalistic conditions. For this type of epidemiological question (one variable observed under two different conditions), the comparative observational study design with statistical analysis to control for confounding, as done here, is a standard method [74, 84–87].

The object of the study was an integrative whole medical system (AM, including physician advice, AM medications, nonmedication treatment, and conventional therapy including antibiotics if necessary). The research questions of the present analysis correspond to the two first stages of a proposed five-stage strategy for assessment of whole medical systems [69]: stage I - description of the system and its treatment modalities (here: use of antibiotics and other medications); stage II - safety of the whole system. Notably, at the stages I-II the whole system is addressed, whereas components of the system are investigated at subsequent stages. In this study, the whole system encompassed not only the “AM therapy proper” (i.e., the consultation with the A-physicians and the treatment prescribed by them) but also the caregivers who had chosen to consult the A-physicians, and the patients. Hence, any differences in antibiotic prescription between A- and C-patients may have been influenced by: [a] the “AM therapy proper”, [b] any specific features of the families choosing treatment by an A-physician (e.g., life-style, motivation, treatment expectations), and [c] general demographic factors and baseline morbidity of the patients.

In the present analysis, adjustment for demographics and baseline morbidity was straightforward, as the relevant variables had been documented (further discussion underneath). The possibilities for adjustment for caregiver characteristics were, however, limited to two variables: The first variable, caregivers' confidence in the physicians' professional skills, was higher in the A-group, but that could not explain the difference in antibiotic description (additional adjustment for this variable lead to even larger differences). The second variable was the question “Do you have confidence that the treatment your child will receive will solve his/her medical problem?”, which was answered by “yes” in all but two cases and which therefore could not be used for adjustment. Possibly, the dichotomised response possibility of “yes/no” was too simplistic and insensitive to more gradual differences in treatment expectations. As regards the “AM therapy proper” complex, the dataset allowed for analysis of one therapy factor: consultation length, which also could not explain the difference in antibiotic prescription.

Notably, in the stages I-II of a system evaluation, these limitations are common and are accepted; main focus of interest are the characteristics of the entire system as such, whereas the disentanglement of “pure” treatment effects versus “nonspecific” effects of expectations, caregiver behaviour, and so forth comes second. This affects the generalisability of our findings: the results of this analysis apply to patients of caregivers who consult A-physicians. One cannot infer from the results to what extent antibiotics and analgesics might be avoided in children who usually receive conventional care, if they should be offered AM treatment. For example, the AM approach may require more active engagement than conventional therapy, such as frequent dosing of medication and extended nursing with application of compresses and footbaths [63, 64]. For many caregivers, this may not be feasible or acceptable.

On the other hand, the group of caregivers willing to engage in a treatment strategy for childhood RTI/OM relying less on antibiotics, is not necessarily limited to people with a strong attachment to AM or complementary therapy: The caregivers can visit A-physicians with their children for different reasons, such as geographical proximity, recommendation from family or friends, dissatisfaction with another physician, or because they deliberately choose AM or complementary therapy. This question was not documented in the study, so it could not be further assessed. Notably, in German-speaking countries, the AM approach to RTI/OM in children is well-known; it is described in the standard German-language physicians' textbooks for anthroposophic [88] and integrative paediatrics [89], respectively as well as in several parents guidebooks to complementary treatment for children [90, 91], one of which has been sold in more than 1 million copies [91].

In this study, medication prescriptions were documented by the physicians at baseline and by caregivers in telephone interviews during follow-up. Physicians' prescription data were checked with the patient files during monitor visits and should therefore be accurate. Patient documentation of medications prescribed during follow-up as well as compliance with prescription was not subject to monitoring; therefore, biased reporting of antibiotic prescription and use during follow-up cannot be excluded. Since the interviewers were not blinded towards the treatment setting, reporting bias on part of them can also not be excluded. However, this bias seems unlikely, because none of the interviewers had any financial or personal ties to any treatment regimen or any physician [53].

C-patients had a much higher use of antibiotics than A-patients, but this cannot explain the somewhat slower improvement and recovery of C-patients. If antibiotics should have detrimental short term effects on RTI and OM in children, one would expect the antibiotic groups in randomised trials of RTI/OM to have slower improvement and recovery, compared to the control groups. This is not the case, placebo-controlled randomised trials of RTI/OM in children consistently show similar or quicker resolution in the antibiotic groups, compared to the placebo groups [9–12].

A total of 4.5% of the A-patients and 8.1% of the C-patients had no antibiotic prescription on day 0 but had a prescription on days 1–28. Whether this was due to a delayed prescription at baseline or due to an emerging need for antibiotic treatment during follow-up cannot be assessed, as the study documentation did not discern between immediate and delayed prescription.

As in all comparative epidemiological studies there is the issue of confounding by baseline differences. In this study sample, some relevant morbidity variables differed between the groups at baseline but were adjusted for (chief complaint of ear pain, duration of chief complaint, symptom intensity, and concomitant respiratory disorders). The groups also differed regarding participating countries, which was not adjusted for in this paediatric subgroup analysis since the analysed sample had less than ten C-patients in three of the five countries. However, exclusion of the children from the USA (with no C-patients) yielded similar results in this paediatric sample. Furthermore, in the full sample of adults and children, the adjustment for country had no perceptible influence on the outcomes [53]. A number of other factors did not differ between the groups (including gender, age, chief complaints of sore throat or cough, recurrent chief complaints, and fever). Nonetheless, our adjustment models are, like all statistical adjustment models, imperfect representations of reality [92], and residual confounding cannot be ruled out. Furthermore, since this is a secondary subgroup analysis, results are hypothesis-generating, not confirmative. On the other hand, the differences in antibiotic and analgesic prescription seem much too large (OR >6 for no antibiotics and OR >12 for no analgesics in all models) to be explained by confounding.

To sum up: This analysis was a naturalistic comparison of two different treatment systems applied under routine conditions. The research questions addressed features of AM treatment for children whose caregivers had chosen to consult physicians offering this therapy. The analysis showed, after considering relevant confounders and adjusting for them as far as possible, that children treated by A-physicians used much less antibiotics and analgesics/antipyretics. The AM treatment entailed no safety problem and was not associated with delayed short-term recovery. On the contrary, A-patients had somewhat quicker short-term resolution than C-patients. However, the study and the present analysis cannot tell us to what extent antibiotics and analgesics could have been avoided among the C-patients, if they had been offered AM treatment. Likewise, an effectiveness evaluation on the level of individual AM medications was beyond the scope of this system evaluation study [83, 93].

### 4.3. Interpretation, Relation to Previously Published Work

This analysis showed a very low use of antibiotics and analgesics in children treated for RTI/OM by AM physicians, without detrimental effects. The study data were collected in 1999-2000, and antibiotic use for RTI/OM in children has since then been reduced. However, naturalistic studies of children with RTI/OM published in the period 2006–2012 still show antibiotic prescription rates 6 to 29 times higher than in the A-patients with corresponding diagnoses (Figure 1).

The very low antibiotic use in A-patients in this study is consistent with findings from a large multicentre prospective observational study in AM primary care settings in Germany, in which antibiotics were prescribed to 5.8% of 14,945 cases with upper RTI in 8,900 children on days 0–15 [51]. Similarly, two smaller prospective observational studies from single AM practices reported very low antibiotic prescription rates in mixed groups of adults and children (1.5% of 132 cases with OM with fever [48], 7.5% of 329 cases with suppurative tonsillitis [50]), without safety problems. Low use of antibiotics and analgesics has also been found in children with an anthroposophic life-style (children attending Waldorf schools [94, 95] and children attending an AM child welfare centre [96]).

Antibiotic [17–24] and analgesic/antipyretic [38–41] use in early childhood are putative risk factors for allergic and autoimmune diseases. In well-controlled paediatric epidemiological studies, an anthroposophic life-style (including low use of antibiotics and antipyretics and a diet containing fermented vegetables) was associated with a reduced risk for atopic disease [94, 95] and with differences in intestinal bacterial flora [96].

### 4.4. Implications for Practice and Research

According to the results of this analysis, AM treatment for RTI/OM in children whose caregivers choose this therapy is safe and associated with a very low use of antibiotics and analgesics, compared to current practice in conventional primary care settings. This raises the issue if some elements of the AM approach to RTI/OM might also be utilized in conventional medical settings. In this context, future studies of AM therapy for RTI/OM in children in order to further characterise the components of AM treatment, such as the type of external applications, other advice given to caregivers, and the AM medications, could be helpful. Other topics of interest include long-term treatment of recurrent RTI/OM and health economics. Randomised trials are difficult to conduct in AM settings, chiefly because the physician-patient-relationship is disturbed by randomisation and because of strong therapy preferences [53, 97]. However, some components of AM therapy for RTI/OM are standardised and can also be prescribed by physicians without extensive knowledge of AM principles (e.g., individual AM medications [98, 99] or nonmedication therapies [100]). Study results would seem to warrant a testing of such therapy components also in non-AM settings.

## 5. Conclusions

This analysis from a prospective observational study under routine primary care conditions showed a very low use of antibiotics and analgesics/antipyretics in children treated for RTI/OM by physicians offering AM therapy, compared to current practice in conventional therapy settings (antibiotics prescribed to 5% versus 26% of A- and C-patients, respectively, during days 0–28; antipyretics prescribed to 3% versus 26%). The AM treatment entailed no safety problem and was not associated with delayed short-term recovery. These differences could not explained by differences in demographics or baseline morbidity. The low antibiotic use is consistent with findings from other studies of paediatric RTI/OM in AM settings.

Notably, the results of this analysis apply to patients of caregivers who consult A-physicians. One cannot infer from the results to what extent antibiotics and analgesics might be avoided in children who usually receive conventional care, if they should be offered AM treatment.

## Supplementary Material

Supplementary Table 1: Prescription of six most common Anatomical Therapeutic Chemical drug groups.
Supplementary Table 2: Treatment outcome on Days 7, 14 and 28.
Supplementary Table 3: Subgroup analysis of main outcomes according to chief complaint and age.
Supplementary Table 4: Sensitivity analysis [a]: Odds ratios for main outcomes after exclusion of patients from the USA.
Supplementary Table 5: Sensitivity analyses [b-d]: Odds ratios for main outcomes after substitution of one independent variable for another.
Supplementary Table 6: Sensitivity analysis [e]: Odds ratios for main outcomes, adjustment also for previous treatment by the study physician.
Supplementary Table 7: Sensitivity analysis [f]: Odds ratios for main outcomes, adjustment also for body mass index.
Supplementary Table 8: Sensitivity analysis [g]: Odds ratios for main outcomes, adjustment also for household size.
Supplementary Table 9: Sensitivity analysis [h]: Odds ratios for main outcomes, adjustment also for household income.
Supplementary Table 10: Sensitivity analyses [i-k]: Odds ratios for main outcomes, adjustment also for caregiver's confidence in physician's professional skill and consultation length.
Supplementary Figure 1: Patient recruitment and follow-up telephone interviews.

## Figures and Tables

**Table 1:** Demographics.

Item	Anthroposophy group (*N* = 443)		Conventional group (*N* = 86)		*P* value
	*N*	%	*N*	%	
Country					<0.001
Austria	91	20.5%	4	4.7%	
Germany	265	59.8%	9	10.5%	
Netherlands	44	9.9%	52	60.5%	
United Kingdom	21	4.7%	21	24.4%	
United States	22	5.0%	0	0.0%	
Male gender	234	52.8%	47	54.7%	0.814
Caucasian race/ethnicity	350/364	96.2%	77/78	98.7%	0.487
Age					0.847
<2 years	89	20.1%	17	19.8%	
2–5 years	196	44.2%	37	43.0%	
6–17 years	158	35.7%	32	33.3%	
Body mass index (mean ± SD)	16.1 ± 2.7		16.5 ± 2.9		0.300
Persons in household (mean ± SD)	3.8 ± 0.9		4.0 ± 0.8		0.051
Total annual household income	*N* = 213		*N* = 38		0.651
<15,000 €	40	18.8%	4	10.5%	
15,000–29,999 €	57	26.8%	11	28.9%	
30,000–44,999 €	64	30.0%	14	36.8%	
45,000–59,999 €	23	10.8%	8	21.1%	
60,000–74,999 €	20	9.4%	0	0.0%	
≥75,000 €	9	4.2%	1	2.6%	
Previous treatment by physician	333/360	92.5%	72/78	92.3%	1.000

**Table 2:** Comparison to other studies: search strategies.

*Search strategy for PubMed: Respiratory infections *	
((“English”[Language]) OR “German”[Language])) AND “antibacterial agents”[MeSH Terms] AND “respiratory tract infections”[MeSH Terms] AND (“2006”[Date-Publication]: “2012”[Date-Publication]) NOT “randomized controlled trial”[Publication Type] NOT pneumonia[Title] NOT tuberculosis[Title] NOT “case reports”[Publication Type] NOT lower respiratory tract[Title] NOT adult[Title] NOT urinary tract[Title]	

*Search strategy for PubMed: Otitis media *	
(((“English”[Language]) OR (“German”[Language])) AND (Otitis media, suppurative [MeSH Terms]) AND (“2006”[Publication Date]: “2012”[Publication Date]) NOT (“randomized controlled trial”[Publication Type]))	

*Search strategy for Google Scholar *	
allintitle: [“antibiotic use” OR “antibiotic prescription”] + [children OR child OR pediatric OR paediatric]	
Years 2006–2012	

**Table 3:** Comparison to other studies: diagnosis groups.

Diagnosis group	This study	Other studies
1	Sore throat	Sore throat/pharyngitis/tonsillitis (excluding tonsillar hypertrophy)

2	Cough	Cough/tracheitis/bronchitis

3	Ear pain	Ear pain/otitis media (excluding otitis media with effusion)

4	All patients: Sore throat, cough or ear pain	Respiratory tract infection/upper respiratory tract infection with or without otitis media (excluding pneumonia, cystic fibrosis, and tuberculosis)

**Table 4:** Disease status at baseline, consultation length.

Item	Anthroposophy group (*N* = 443)		Conventional group (*N* = 86)		*P*-value
	*N*	%	*N*	%	
Chief complaint					
Sore throat	98	22.1%	14	16.3%	
Cough	215	48.5%	35	40.7%	0.001
Ear pain	130	29.3%	37	43.0%	
Duration of chief complaint					
0–≤24 h	160	36.1%	18	20.9%	
>24 h–≤48 h	121	27.3%	22	25.6%	0.002
>2 days–≤7 days	161	36.3%	46	53.5%	
Severe or very severe intensity of chief complaint					
(i) all patients	284	64.3%	44	51.2%	0.028
(ii) chief complaint ear pain	93/130	71.5%	18/37	48.6%	0.017
Fever ≥ 38.5°C	85	19.2%	13	15.1%	0.449
Symptom score (0–4, mean ± SD)	1.3 ± 0.7		1.0 ± 0.6		0.011
Diagnosis of chief complaint					0.002
Otitis media	114	25.7%	29	33.7%	0.144
Laryngitis/tracheitis/bronchitis	150	33.9%	18	20.9%	0.022
Pharyngitis/tonsillitis	103	23.3%	12	14.0%	0.063
Common cold/upper respiratory tract infection	35	7.9%	16	18.6%	0.004
Other	41	9.3%	11	12.8%	
Physician's confidence in diagnosis (0–10, mean ± SD)	9.4 ± 1.0		9.1 ± 1.2		0.136
(i) based on clinical examination	433	97.7%	79	91.9%	0.023
(ii) based on symptoms alone	10	2.3%	7	8.1%	
Chief complaint episode within last 12 months	271	61.2%	42	48.8%	0.053
Concomitant disease present	139	31.4%	23	26.7%	0.444
Disease of respiratory system	40	9.0%	17	19.8%	0.007
Medication use for concomitant disease	67	15.1%	12	14.0%	0.870
Anti-asthmatics	4	0.9%	7	8.1%	<0.001
Caregiver's confidence in physician's professional skill	*N* = 361		*N* = 77		
Extremely	272	75.3%	33	42.9%	
Quite a bit	82	22.7%	32	41.6%	<0.001
Moderately	5	1.4%	11	14.3%	
Slightly or not at all	2	0.6%	1	0.3%	
Does caregiver have confidence that the treatment will solve the medical problem? (yes/no)-yes	357/358	99.7%	74/75	98.7%	0.317
Did caregiver have freedom to choose this physician? (yes/no)-yes	352/362	97.2%	53/58	91.4%	0.0424
Consultation length					
<5 min	6	1.4%	15	17.4%	
>5–≤15 min	226	51.0%	68	79.1%	<0.001
>15–≤30 min	208	47.0%	3	3.5%	
>30–≤60 min	3	0.7%	0	0.0%	

**Table 5:** Comparison other studies: study characteristics.

Study	Land	Design	*N*	Age years	Diagnoses/subgroups	Reference
This study	*AT, DE, NL, UK, US *	*POCS *	*529 *	*0*–*17 *	Sore throat/ear pain/Cough	
Abbas et al., 2010	DE	RDA	21,564	2–17	Respiratory infection/nonsupportive OM/supportive OM	[75]
Ashe et al., 2006	US	POCS	720	0.5–12	Symptoms of respiratory illness or OM	[76]
Ashworth et al., 2006	UK	RDA	>100,000	1–16	Sore throat/ear infection/tracheitis or bronchitis	[77]
Chung et al., 2007	UK	POCS	119	0.5–12	Suspected respiratory infection or OM	[78]
Coco et al., 2010	US	POCS	1,114	0.5–12	OM	[79]
Harnden et al., 2007	UK	POCS	425	0.5–12	Cough and fever, “more than a simple cold,” physician considered prescribing antibiotics	[80]
Meropol et al., 2009	UK	RDA	>400,000	1–17	Nonspecific respiratory infection (excluding OM and sinusitis)	[7]
Plasschaert et al., 2006	NL	RDA	>8,000	0–13	OM	[2]
Uijen et al., 2011	NL	RDA	>50,000	0–17	Tonsillitis/OM	[81]
van Deursen et al., 2012	NL	RDA	>5,000	2–17	Respiratory infection	[8]
Zuckerman et al., 2007	US	RDA	20,213	0–5	Upper respiratory tract infection	[82]

OM: otitis media. POCS: prospective observational cohort study. RDA: retrospective database analysis.

**Table 6:** Odds ratios for main outcomes.

Outcome	Outcome rate					Unadjusted odds ratio (A- vs. C-)		Adjusted odds ratio (A- vs. C-)		
	A-Group		C-Group					
	*N* = 443		*N* = 86		*P* value	OR	(95%-CI)	OR	(95%-CI)	
	*N*	%	*N*	%				
No antibiotics days 0–28	421	95.0%	64	74.4%	<0.001	6.58	(3.45–12.56)	6.33	3.17	12.64
No analgesics days 0–28	429	96.8%	64	74.4%	<0.001	10.53	(5.13–21.63)	12.11	5.50	26.69
First improvement ≤24 hours	168	37.9%	17	19.8%	0.003	2.48	(1.41–4.36)	2.57	1.40	4.72
First improvement ≤3 days	358	80.8%	57	66.3%	0.019	2.14	(1.29–3.55)	1.88	1.10	3.23
Response on day 7	373	84.2%	54	62.3%	<0.001	3.16	(1.90–5.24)	3.34	1.93	5.79
Response on day 14	417	94.1%	74	86.0%	0.020	2.60	(1.26–5.38)	2.53	1.18	5.44
Recovery on day 7	155	35.0%	26	30.2%	0.457	1.24	(0.75–2.05)	1.19	0.68	2.07
Recovery on day 14	322	72.7%	50	58.1%	0.010	1.92	(1.19–3.09)	1.98	1.18	3.32
Very satisfied with treatment^*^	316	71.3%	36	36.0%	<0.001	3.46	(2.15–5.56)	4.15	2.50	6.86
Choosing this therapy again^*^	435	98.2%	65	75.6%	<0.001	17.57	(7.47–41.31)	18.92	7.65	46.81

Main outcomes: outcome rates, unadjusted odds ratios (OR) with 95% confidence intervals, and odds ratios after multiple logistic regression analysis, adjusting for gender, age, chief complaint, duration of complaint, complaint episode within last 12 months, baseline symptom score, and concomitant disease present at baseline. Odds ratio >1 indicates better outcome in A-group. ^*^at all available follow-ups.

## Abbreviations

A- and C-groups, A- and C-patients: Patients treated by anthroposophic and conventional physicians, respectively
AM: Anthroposophic medicine
CI: Confidence interval
IIPCOS: International Integrative Primary Care Outcomes Study
OM: Otitis media
OR: Odds ratio
RTI: Respiratory tract infections
SA: Sensitivity analysis.

## References

[1] Cherry D. K., Burt C. W., Woodwell D. A. (2003). National Ambulatory Medical Care Survey: 2001 summary. *Advance Data*.

[2] Plasschaert A. I. O., Rovers M. M., Schilder A. G. M., Verheij T. J. M., Hak E. (2006). Trends in doctor consultations, antibiotic prescription, and specialist referrals for otitis media in children: 1995–2003. *Pediatrics*.

[3] Andre M., Vernby Å., Odenholt I., Stålsby Lundborg C., Axelsson I., Eriksson M., Runehagen A., Schwan Å., Molstad S. (2008). Diagnosis-prescribing surveys in 2000, 2002 and 2005 in Swedish general practice: consultations, diagnosis, diagnostics and treatment choices. *Scandinavian Journal of Infectious Diseases*.

[4] de Jong J., van den Berg P. B., de Vries T. W., de Jong-van den Berg L. T. W. (2008). Antibiotic drug use of children in the Netherlands from 1999 till 2005. *European Journal of Clinical Pharmacology*.

[5] Thompson P. L., Gilbert R. E., Long P. F., Saxena S., Sharland M., Wong I. C. K. (2008). Has UK guidance affected general practitioner antibiotic prescribing for otitis media in children?. *Journal of Public Health*.

[6] Thompson P. L., Spyridis N., Sharland M. (2009). Changes in clinical indications for community antibiotic prescribing for children in the UK from 1996 to 2006: will the new NICE prescribing guidance on upper respiratory tract infections just be ignored?. *Archives of Disease in Childhood*.

[7] Meropol S. B., Chen Z., Metlay J. P. (2009). Reduced antibiotic prescribing for acute respiratory infections in adults and children. *The British Journal of General Practice*.

[8] van Deursen A. M. M., Verheij T. J. M., Rovers M. M. (2012). Trends in primary-care consultations, comorbidities, and antibiotic prescriptions for respiratory infections in the Netherlands before implementation of pneumococcal vaccines for infants. *Epidemiology & Infection*.

[9] Spinks A., Glasziou P. P., del Mar C. B. (2013). Antibiotics for sore throat. *The Cochrane Database of Systematic Reviews*.

[10] Smith S. M., Fahey T., Smucny J., Becker L. A. (2014). Antibiotics for acute bronchitis. *Cochrane Database of Systematic Reviews*.

[11] Kenealy T., Arroll B. (2013). Antibiotics for the common cold and acute purulent rhinitis. *The Cochrane Database of Systematic Reviews*.

[12] Venekamp R. P., Sanders S., Glasziou P. P., Del Mar C. B., Rovers M. M. (2013). Antibiotics for acute otitis media in children. *Cochrane Database of Systematic Reviews*.

[13] Rosenfeld R. M., Kay D. (2003). Natural history of untreated otitis media. *Laryngoscope*.

[14] Petersen I., Johnson A. M., Islam A., Duckworth G., Livermore D. M., Hayward A. C. (2007). Protective effect of antibiotics against serious complications of common respiratory tract infections: retrospective cohort study with the UK General Practice Research Database. *The British Medical Journal*.

[15] Wise R., Hart T., Cars O., Streulens M., Helmuth R., Huovinen P., Sprenger M. (1998). Antimicrobial resistance. *The British Medical Journal*.

[16] Bezáková N., Damoiseaux R. A. M. J., Hoes A. W., Schilder A. G. M., Rovers M. M. (2009). Recurrence up to 3.5 years after antibiotic treatment of acute otitis media in very young Dutch children: survey of trial participants. *The British Medical Journal*.

[17] Marra F., Marra C. A., Richardson K., Lynd L. D., Kozyrskyj A., Patrick D. M., Bowie W. R., Fitzgerald J. M. (2009). Antibiotic use in children is associated with increased risk of asthma. *Pediatrics*.

[18] Farquhar H., Stewart A., Mitchell E., Crane J., Eyers S., Weatherall M., Beasley R. (2010). The role of paracetamol in the pathogenesis of asthma. *Clinical and Experimental Allergy*.

[19] Risnes K. R., Belanger K., Murk W., Bracken M. B. (2011). Antibiotic exposure by 6 months and asthma and allergy at 6 years: findings in a cohort of 1,401 US children. *The American Journal of Epidemiology*.

[20] Penders J., Kummeling I., Thijs C. (2011). Infant antibiotic use and wheeze and asthma risk: a systematic review and meta-analysis. *European Respiratory Journal*.

[21] Schmitt J., Schmitt N. M., Kirch W., Meurer M. (2010). Early exposure to antibiotics and infections and the incidence of atopic eczema: a population-based cohort study. *Pediatric Allergy and Immunology*.

[22] Shaw S. Y., Blanchard J. F., Bernstein C. N. (2010). Association between the use of antibiotics in the first year of life and pediatric inflammatory bowel disease. *The American Journal of Gastroenterology*.

[23] Shaw S. Y., Blanchard J. F., Bernstein C. N. (2011). Association between the use of antibiotics and new diagnoses of Crohn's disease and ulcerative colitis. *American Journal of Gastroenterology*.

[24] Hviid A., Svanström H., Frisch M. (2011). Antibiotic use and inflammatory bowel diseases in childhood. *Gut*.

[25] Communication from The Commission to the European Parliament and the Council (2011). *Action Plan against the Rising Threats from Antimicrobial Resistance*.

[26] Majeed A., Harris T. (1997). Acute otitis media in children. *The British Medical Journal*.

[27] Dowell S. F., Marcy S. M., Phillips W. R., Gerber M. A., Schwartz B. (1998). Principles of judicious use of antimicrobial agents for pediatric upper respiratory tract infections. *Pediatrics*.

[28] Gonzales R., Bartlett J. G., Besser R. E., Cooper R. J., Hickner J. M., Hoffman J. R., Sande M. A. (2001). Principles of appropriate antibiotic use for treatment of acute respiratory tract infections in adults: background, specific aims, and methods. *Annals of Internal Medicine*.

[29] Hirschmann J. V. (2002). Antibiotics for common respiratory tract infections in adults. *Archives of Internal Medicine*.

[30] Mölstad S. (2003). Reduction in antibiotic prescribing for respiratory tract infections is needed!. *Scandinavian Journal of Primary Health Care*.

[31] Andrews T., Thompson M., Buckley D. I., Heneghan C., Deyo R., Redmond N., Lucas P. J., Blair P. S., Hay A. D. (2012). Interventions to influence consulting and antibiotic use for acute respiratory tract infections in children: a systematic review and Meta-Analysis. *PLoS ONE*.

[32] Thibeault R., Gilca R., Côté S., de Serres G., Boivin G., Déry E. P. (2007). Antibiotic use in children is not influenced by the result of rapid antigen detection test for the respiratory syncytial virus. *Journal of Clinical Virology*.

[33] Spiro D. M., Tay K.-Y., Arnold D. H., Dziura J. D., Baker M. D., Shapiro E. D. (2006). Wait-and-see prescription for the treatment of acute otitis media: a randomized controlled trial. *The Journal of the American Medical Association*.

[34] Spurling G. K., Del Mar C. B., Dooley L., Foxlee R. (2007). Delayed antibiotics for respiratory infections. *Cochrane Database of Systematic Reviews*.

[35] Siegel R. M. (2010). Acute otitis media guidelines, antibiotic use, and shared medical decision-making. *Pediatrics*.

[36] Schulman C. I., Namias N., Doherty J. (2005). The effect of antipyretic therapy upon outcomes in critically ill patients: a randomized, prospective study. *Surgical Infections*.

[37] Nourjah P., Ahmad S. R., Karwoski C., Willy M. (2006). Estimates of acetaminophen (paracetamol)-associated overdoses in the United States. *Pharmacoepidemiology and Drug Safety*.

[38] Beasley R., Clayton T., Crane J. (2008). Association between paracetamol use in infancy and childhood, and risk of asthma, rhinoconjunctivitis, and eczema in children aged 6-7 years: analysis from phase three of the ISAAC programme. *The Lancet*.

[39] Bakkeheim E., Mowinckel P., Carlsen K. H., Håland G., Carlsen K. C. L. (2011). Paracetamol in early infancy: the risk of childhood allergy and asthma. *Acta Paediatrica*.

[40] Wennergren G. (2011). Paracetamol—accumulating reports of an association with allergy and asthma. *Acta Paediatrica*.

[41] Amberbir A., Medhin G., Alem A., Britton J., Davey G., Venn A. (2011). The role of acetaminophen and geohelminth infection on the incidence of wheeze and eczema: a longitudinal birth-cohort study. *American Journal of Respiratory and Critical Care Medicine*.

[42] Sarrell E. M., Mandelberg A., Cohen H. A. (2001). Efficacy of naturopathic extracts in the management of ear pain associated with acute otitis media. *Archives of Pediatrics and Adolescent Medicine*.

[43] Sarrell E. M., Cohen H. A., Kahan E. (2003). Naturopathic treatment for ear pain in children. *Pediatrics*.

[44] Wustrow T. P. U. (2005). Naturheilkundliche Therapie der akuten Otitis media. Eine Alternative zum primären Antibiotikaeinsatz. Naturopathic therapy for acute otitis media. An alternative to the primary use of antibiotics. *HNO*.

[45] Friese K.-H., Kruse S., Lüdtke R., Moeller H. (1997). The homoeopathic treatment of otitis media in children—comparisons with conventional therapy. *International Journal of Clinical Pharmacology and Therapeutics*.

[46] Jacobs J., Springer D. A., Crothers D. (2001). Homeopathic treatment of acute otitis media in children: a preliminary randomized placebo-controlled trial. *Pediatric Infectious Disease Journal*.

[47] Haidvogl M., Riley D. S., Heger M. (2007). Homeopathic and conventional treatment for acute respiratory and ear complaints: a comparative study on outcome in the primary care setting. *BMC Complementary and Alternative Medicine*.

[48] Büttner G. (1973). Die Behandlung der akuten Otitis media ohne Sulfonamide und Antibiotika in einer Allgemeinpraxis [Treatment of acute otitis media without sulphonamides and antibiotics in general practice]. *Beiträge zu einer Erweiterung der Heilkunst*.

[49] Fried R. (1990). My experiences with acute and chronic middle ear inflammations in children. *Journal of Anthroposophical Medicine*.

[50] Husemann F. (1998). Scharlach und eitrige Angina in zehn Jahren Praxis [Scarlet fever and suppurative tonsillitis-a ten-year practice study]. *Der Merkurstab*.

[51] Jeschke E., Lüke C., Ostermann T., Tabali M., Hübner J., Matthes H. (2007). Prescribing practices in the treatment of upper respiratory tract infections in anthroposophic medicine. *Forschende Komplementarmedizin*.

[52] Cosby J. L., Francis N., Butler C. C. (2007). The role of evidence in the decline of antibiotic use for common respiratory infections in primary care. *Lancet Infectious Diseases*.

[53] Hamre H. J., Fischer M., Heger M., Riley D., Haidvogl M., Baars E., Bristol E., Evans M., Schwarz R., Kiene H. (2005). Anthroposophic vs. conventional therapy of acute respiratory and ear infections: a prospective outcomes study. *Wiener Klinische Wochenschrift*.

[54] Lasky T. (2009). Estimates of pediatric medication use in the United States: current abilities and limitations. *Clinical Therapeutics*.

[55] Grossman Z., del Torso S., Hadjipanayis A., van Esso D., Drabik A., Sharland M. (2012). Antibiotic prescribing for upper respiratory infections: European primary paediatricians' knowledge, attitudes and practice. *Acta Paediatrica*.

[56] Joseph M. P., Craig P. J., Caldwell D. P. (2013). Clinical trials in children. *British Journal of Clinical Pharmacology*.

[57] Kozyrskyj A. L., Carrie A. G., Mazowita G. B., Lix L. M., Klassen T. P., Law B. J. (2004). Decrease in antibiotic use among children in the 1990s: not all antibiotics, not all children. *Canadian Medical Association Journal*.

[58] Steiner R., Wegman I. (2000). *Extending Practical Medicine: Fundamental Principles Based on the Science of the Spirit*.

[59] Kienle G. S., Albonico H. U., Baars E., Hamre H. J., Zimmermann P., Kiene H. (2013). Anthroposophic medicine: an integrative medical system originating in europe. *Global Advances in Health and Medicine*.

[60] Husemann F., Wolff O. (1987). *The Anthroposophical Approach to Medicine*.

[61] Evans M., Rodger I. (1992). *Anthroposophical Medicine: Healing for Body, Soul and Spirit*.

[62] Ritchie J., Wilkinson J., Gantley M., Feder G., Carter Y., Formby J. (2001). *A Model of Integrated Primary Care: Anthroposophic Medicine*.

[63] Lang-Roth R., Rietschel E., van Koningsbruggen S., Schwarz R., Schönau E., Naumann E. G., Längler A., Beuth J. (2005). Erkrankungen des Hals-Nasen-Ohren-Bereiches [Ear-nose-throat diseases]. *Pädiatrie Integrativ. Konventionelle und Komplementäre Therapie [Integrative Pediatrics. Conventional and Complementary Treatment]*.

[64] Soldner G., Stellmann H. M. (2014). *Individual Paediatrics: Physical, Emotional and Spiritual Aspects of Diagnosis and Counseling. Anthroposophic-Homeopathic Therapy*.

[65] (2013). *Anthroposophic Pharmaceutical Codex APC*.

[66] Buchholzer M.-L., Werner C., Knoess W. (2014). Current concepts on integrative safety assessment of active substances of botanical, mineral or chemical origin in homeopathic medicinal products within the European regulatory framework. *Regulatory Toxicology and Pharmacology*.

[67] Kienle G. S., Kiene H., Albonico H. U. (2006). *Anthroposophic Medicine: Effectiveness, Utility, Costs, Safety*.

[68] Hamre H. J., Kiene H., Kienle G. S. (2009). Clinical research in anthroposophic medicine. *Alternative Therapies in Health and Medicine*.

[69] Fønnebø V., Grimsgaard S., Walach H. (2007). Researching complementary and alternative treatments—the gatekeepers are not at home. *BMC Medical Research Methodology*.

[70] Shrout P. E., Fleiss J. L. (1979). Intraclass correlations: uses in assessing rater reliability. *Psychological Bulletin*.

[71] Hosmer D. W., Lemeshow S. (2000). *Applied Logistic Refression*.

[72] Menard S. (2002). *Applied Logistic Regression Analysis*.

[73] Hamre H. J., Glockmann A., Tröger W., Kienle G. S., Kiene H. (2008). Assessing the order of magnitude of outcomes in single-arm cohorts through systematic comparison with corresponding cohorts: an example from the AMOS study. *BMC Medical Research Methodology*.

[74] von Elm E., Egger M., Altman D. G., Pocock S. J., Gøtzsche P. C., Vandenbroucke J. P. (2007). Strengthening the reporting of observational studies in epidemiology (STROBE) statement: guidelines for reporting observational studies. *British Medical Journal*.

[75] Abbas S., Ihle P., Heymans L., Küpper-Nybelen J., Schubert I. (2010). Differences in antibiotic prescribing between general practitioners and pediatricians in Hesse, Germany. *Deutsche Medizinische Wochenschrift*.

[76] Ashe D., Patrick P. A., Stempel M. M., Shi Q., Brand D. A. (2006). Educational posters to reduce antibiotic use. *Journal of Pediatric Health Care*.

[77] Ashworth M., Charlton J., Latinovic R., Gulliford M. (2006). Age-related changes in consultations and antibiotic prescribing for acute respiratory infections, 1995–2000. Data from the UK General Practice Research Database. *Journal of Clinical Pharmacy and Therapeutics*.

[78] Chung A., Perera R., Brueggemann A. B., Elamin A. E., Harnden A., Mayon-White R., Smith S., Crook D. W., Mant D. (2007). Effect of antibiotic prescribing on antibiotic resistance in individual children in primary care: prospective cohort study. *British Medical Journal*.

[79] Coco A., Vernacchio L., Horst M., Anderson A. (2010). Management of acute otitis media after publication of the 2004 AAP and AAFP clinical practice guideline. *Pediatrics*.

[80] Harnden A., Perera R., Brueggemann A. B. (2007). Respiratory infections for which general practitioners consider prescribing an antibiotic: a prospective study. *Archives of Disease in Childhood*.

[81] Uijen J. H., Bindels P. J., Schellevis F. G., van der Wouden J. C. (2011). ENT problems in Dutch children: trends in incidence rates, antibiotic prescribing and referrals 2002–2008. *Scandinavian Journal of Primary Health Care*.

[82] Zuckerman I. H., Perencevich E. N., Harris A. D. (2007). Concurrent acute illness and comorbid conditions poorly predict antibiotic use in upper respiratory tract infections: a cross-sectional analysis. *BMC Infectious Diseases*.

[83] Hamre H. J., Glockmann A., Fischer M., Riley D. S., Baars E., Kiene H. (2007). Use and safety of anthroposophic medications for acute respiratory and ear infections: a prospective cohort study. *Drug Target Insights*.

[84] Akkerman A. E., Kuyvenhoven M. M., van der Wouden J. C., Verheij T. J. M. (2005). Prescribing antibiotics for respiratory tract infections by GPs: management and prescriber characteristics. *British Journal of General Practice*.

[85] Salomon J., Sommet A., Bernède C., Tonéatti C., Carbon C., Guillemot D. (2008). Antibiotics for nasopharyngitis are associated with a lower risk of office-based physician visit for acute otitis media within 14 days for 3- to 6-year-old children. *Journal of Evaluation in Clinical Practice*.

[86] Elshout G., Van Ierland Y., Bohnen A. M., De Wilde M., Oostenbrink R., Moll H. A., Berger M. Y. (2013). Alarm signs and antibiotic prescription in febrile children in primary care: an observational cohort study. *British Journal of General Practice*.

[87] Little P., Stuart B., Hobbs F. D. R. (2014). Antibiotic prescription strategies for acute sore throat: a prospective observational cohort study. *The Lancet Infectious Diseases*.

[88] Soldner G., Stellmann H. M. (2011). *Individuelle Pädiatrie: Leibliche, seelische und geistige Aspekte in Diagnostik und Beratung*.

[89] Schönau E., Naumann E. G., Längler A., Beuth J. (2005). *Pädiatrie Integrativ. Konventionelle und Komplementäre Therapie [Integrative Pediatrics. Conventional and Complementary Treatment]*.

[90] Vagedes J., Soldner G. (2008). *Das Kinder-Gesundheitsbuch: Kinderkrankheiten ganzheitlich vorbeugen und heilen*.

[91] Stellmann H. M., Soldner G. (2014). *Kinderkrankheiten Natürlich Behandeln [Natural Treatment of Childhood Diseases]*.

[92] Lindsey J. K. (1997). *Applying Generalized Linear Models*.

[93] Kienle G. S., Albonico H.-U., Fischer L., Frei-Erb M., Hamre H. J., Heusser P., Matthiessen P. F., Renfer A., Kiene H. (2011). Complementary therapy systems and their integrative evaluation. *Explore: The Journal of Science and Healing*.

[94] Alm J. S., Swartz J., Lilja G., Scheynius A., Pershagen G. (1999). Atopy in children of families with an anthroposophic lifestyle. *The Lancet*.

[95] Flöistrup H., Swartz J., Bergström A., Alm J. S., Scheynius A., Van Hage M., Waser M., Braun-Fahrländer C., Schram-Bijkerk D., Huber M., Zutavern A., Von Mutius E., Üblagger E., Riedler J., Michaels K. B., Pershagen G., Pershagen G., Alfvén T., Alm J., Bergström A., Engstrand L., Flöistrup H., Van Hage M., Håkansson N., Lilja G., Nyberg F., Scheynius A., Swartz J., Wickman M., Braun-Fahrländer C., Waser M., Sennhauser F., Lauener R., Wildhaber J., Möller A., Brunekreef B., Schram-Bijkerk D., Doekes G., Boeve M., Douwes J., Matze M., Benz M. R., Budde J., Ege M., Riedler J., Eder W., Üblagger E., Weiss G., Schreuer M., Michels K. B. (2006). Allergic disease and sensitization in Steiner school children. *Journal of Allergy and Clinical Immunology*.

[96] Alm J. S., Swartz J., Björkstén B., Engstrand L., Engström J., Kühn I., Lilja G., Möllby R., Norin E., Pershagen G., Reinders C., Wreiber K., Scheynius A. (2002). An anthroposophic lifestyle and intestinal microflora in infancy. *Pediatric Allergy and Immunology*.

[97] Ziegler R. (2009). Mistletoe preparation iscador: are there methodological concerns with respect to controlled clinical trials?. *Evidence-Based Complementary and Alternative Medicine*.

[98] Meyer U. (2003). Anwendungsbeobachtung WALA Plantago-Bronchialbalsam [Observational study of WALA Plantago Bronchial Balm]. *Der Merkurstab*.

[99] Rother C., Steigerwald P. (2007). Effectiveness and safety of the anthroposophic medicinal product Ferrum phosphoricum comp. for prodromal and manifest influenza. *Der Merkurstab*.

[100] Kienle G. S., Kiene H., Albonico H. U. (2006). *Ulbricht 1991 [Pseudorandomised Comparative Study on the Influence of Body-Temperature Enemas on Fever]. Anthroposophic Medicine: Effectiveness, Utility, Costs, Safety*.

