# Supplementary material for: Antibiotic Use in Children with Acute Respiratory or Ear Infections: Prospective Observational Comparison of Anthroposophic and Conventional Treatment under Routine Primary Care Conditions
Source: Evid Based Complement Alternat Med. 2014 Nov 18;2014:243801. doi: 10.1155/2014/243801 (PMC4251819; doi:10.1155/2014/243801)

Supplementary Table 1 Prescription of six most common Anatomical Therapeutic Chemical drug groups

Anatomical Therapeutic Chemical group	Day 0					Cumulative: Day 0-28				
	Anthroposophy group		Conventional group			Anthroposophy group		Conventional group		
	N	%	N	%	P-value	N	%	N	%	P-value
J01 Antibacterials for systemic use	2	0.5%	15	17,4%	< 0.001	22	5.0%	22	25.6%	< 0.001
N02 Analgesics	8	1.8%	22	25.6%	< 0.001	14	3.2%	22	25.6%	< 0.001
R01 Nasal preparations	82	18.5%	19	22.1%	0.454	89	20.1%	21	24.4%	0.384
R05 Cough and cold preparations	99	22.3%	3	3.5%	< 0.001	109	24.6%	5	5.8%	< 0.001
M01 Anti-inflammatory and antirheumatic products	0	0.0%	3	3.5%	0.004	0	0.0%	3	3.5%	0.004
R06 Antihistamines for systemic use	0	0.0%	7	8.1%	< 0.001	0	0.0%	8	9.3%	< 0.001

Percentage of patients receiving a prescription. Anthroposophy (A-) Group n = 443; Conventional (C-) Group n = 86.

Supplementary Table 2 Treatment outcome on Days 7, 14 and 28

Treatment outcome	Day 7				Day 14				Day 28			
	Anthroposophy group		Conventional group		Anthroposophy group		Conventional group		Anthroposophy group		Conventional group	
	N	%	N	%	N	%	N	%	N	%	N	%
Complete recovery	155	35.0%	26	30.2%	322	72.7%	50	58.1%	388	87.6%	67	77.9%
Major improvement	218	49.2%	28	32.6%	95	21.4%	24	27.9%	38	8.6%	13	15.1%
Slight to moderate improvement	34	7.7%	20	23.3%	19	4.3%	9	10.5%	11	2.5%	5	5.8%
No change	7	1.6%	4	4.7%	4	0.9%	1	1.2%	5	1.1%	1	1.2%
Deterioration	2	0.5%	0	0.0%	1	0.2%	0	0.0%	1	0.2%	0	0.0%
Missing	27	6.1%	8	9.3%	2	0.5%	2	2.3%	0	0.0%	0	0.0%
Total	443	100.0%	86	100.0%	443	100.0%	86	100.0%	443	100.0%	86	100.0%
Response (complete recovery or major improvement)	373	77.1%	54	66.1%	417	89.7%	74	84.4%	426	95.4%	80	95.0%

Last observation carried forward.

Supplementary Table 3 Subgroup analysis of main outcomes according to chief complaint and age

Subgroups	Number of patients		Percentage of patients															
			Day 1 First im-provement		Day 3 First im-provement		Day 7 Major im-provement + Complete recovery		Day 7 Complete recovery		Day 14 Major im-provement + Complete recovery		Day 14 Complete recovery		Days 0-28 No antibiotics		Days 0-28 No analgesics	
	A-	C-	A-	C-	A-	C-	A-	C-	A-	C-	A-	C-	A-	C-	A-	C-	A-	C-
Sore throat	98	14	39.8	28.6	85.7	64.3	91.8	72.9	51.0	42.9	96.9	90.0	85.7	64.3	98.0	78.6	98.0	78.6
Ear pain	130	37	58.5	27.0	84.6	78.4	90.8	70.3	48.5	45.9	96.2	91.9	77.7	73.0	93.1	67.6	93.1	67.6
Cough	215	35	24.7	8.6	76.3	54.3	76.7	57.1	19.5	8.6	91.6	80.0	63.7	40.0	94.9	80.0	94.9	80.0
Age 1-23 months	89	17	29.2	5.9	77.5	64.7	83.1	58.8	24.7	17.6	94.4	76.5	73.0	52.9	92.1	82.4	92.1	82.4
Age 2-5 years	196	37	46.9	37.8	84.7	75.7	87.2	67.6	44.9	37.8	95.4	86.5	76.5	62.2	94.9	78.4	94.9	78.4
Age 6-17 years	158	32	31.6	6.3	77.8	56.3	81.0	59.4	28.5	28.1	92.4	90.6	67.7	56.3	96.8	65.6	96.8	65.6
All patients	443	86	37.9	19.8	80.8	66.3	84.2	62.3	35.0	30.2	94.1	86.0	72.7	58.1	95.5	74.4	95.0	74.4

Cumulative percentage of Anthroposophy group (A-) and Conventional group (C-) with first improvement on Days 1 and 3, with major improvement and complete recovery on Days 7 and 14, respectively, and with no antibiotic and no analgesic prescription, respectively, in subgroups according to chief complaint and age.

Supplementary Table 4 Sensitivity analysis [a]: Odds ratios for main outcomes after exclusion of patients from the USA

Outcome	Unadjusted			Adjusted		
	Odds ratio	95% confidence interval		Odds ratio	95% confidence interval	
		Lower margin	Upper margin		Lower margin	Upper margin
No antibiotics Days 0-28	6.55	3.41	12.59	6.34	3.15	12.77
No analgesics Days 0-28	9.99	4.86	20.53	11.27	5.09	24.94
First improvement ≤ 24 hours	2.41	1.37	4.25	2.45	1.32	4.55
First improvement ≤ 3 days	2.14	1.28	3.55	1.91	1.10	3.29
Response on Day 7	3.31	1.98	5.52	3.60	2.06	6.31
Response on Day 14	2.81	1.34	5.89	2.98	1.35	6.55
Recovery on day 7	1.28	0.77	2.11	1.24	0.71	2.18
Recovery on day 14	2.04	1.26	3.29	2.20	1.30	3.73
Very satisfied with treatment*	4.30	2.64	7.00	3.98	2.39	6.60
Choosing this therapy again*	16.68	7.09	39.23	17.94	7.24	44.48

Main outcomes: unadjusted odds ratios (Anthroposophy group vs. Conventional group) with 95% confidence intervals; odds ratios after multiple logistic regression analysis with adjustment for adjusting for gender, age, chief complaint, duration of complaint, complaint episode within last 12 months, baseline symptom score, concomitant disease present at baseline. Odds ratio > 1 indicates better outcome in Anthroposophy group. Sample restriction to patients from Austria, Germany, The Netherlands, and the UK, n = 507. *At all available follow-ups.

Supplementary Table 5 Sensitivity analyses [b-d]: Odds ratios for main outcomes after substitution of one independent variable for another

Outcome	Unadjusted			Adjusted								
				Sensitivity analysis [b]			Sensitivity analysis [c]			Sensitivity analysis [d]		
	Odds ratio	95% confidence interval		Odds ratio	95% confidence interval		Odds ratio	95% confidence interval		Odds ratio	95% confidence interval	
		Lower margin	Upper margin		Lower margin	Upper margin		Lower margin	Upper margin		Lower margin	Upper margin
No antibiotics Days 0-28	6.58	3.45	12.56	6.35	3.17	12.75	6.39	3.20	12.73	8.94	4.06	19.69
No analgesics Days 0-28	10.53	5.13	21.63	12.30	5.57	27.15	12.21	5.53	26.95	16.16	6.60	39.56
First improvement ≤ 24 hours	2.48	1.41	4.36	2.59	1.40	4.77	2.56	1.39	4.71	2.68	1.46	4.92
First improvement ≤ 3 days	2.14	1.29	3.55	1.94	1.13	3.32	1.91	1.12	3.28	1.85	1.07	3.20
Response on Day 7	3.16	1.90	5.24	3.14	1.81	5.45	3.23	1.87	5.57	3.48	1.99	6.10
Response on Day 14	2.60	1.26	5.32	2.42	1.13	5.21	2.55	1.19	5.45	2.78	1.26	6.14
Recovery on day 7	1.24	0.75	2.05	1.19	0.68	2.06	1.17	0.67	2.04	1.18	0.68	2.07
Recovery on day 14	1.92	1.19	3.09	1.87	1.11	3.14	1.96	1.17	3.29	2.06	1.23	3.45
Very satisfied with treatment*	3.46	2.15	5.56	4.10	2.48	6.79	4.16	2.51	6.90	4.27	2.57	7.10
Choosing this therapy again*	17.57	7.47	41.31	16.72	6.77	41.28	19.90	7.97	49.69	22.36	8.67	57.69

Main outcomes: unadjusted odds ratios (Anthroposophy group vs. Conventional group) with 95% confidence intervals; odds ratios after multiple logistic regression analysis with adjustment for adjusting for gender, age, chief complaint, duration of complaint, complaint episode within last 12 months (SA [b] + SA [d]), number of previous episodes of chief complaint (SA [c]), baseline symptom score (SA [c-d]), baseline severity of chief complaint (SA [b]), concomitant disease present at baseline (SA [b-c]), concomitant respiratory disorder present at baseline (SA [d]). Odds ratio > 1 indicates better outcome in Anthroposophy group. All patients, n = 529. *At all available follow-ups.

Supplementary Table 6 Sensitivity analysis [e]: Odds ratios for main outcomes, adjustment also for previous treatment by the study physician

Outcome	Unadjusted			Adjusted		
	Odds ratio	95% confidence interval		Odds ratio	95% confidence interval	
		Lower margin	Upper margin		Lower margin	Upper margin
No antibiotics Days 0-28	7.92	3.90	16.08	8.83	4.03	19.38
No analgesics Days 0-28	12,89	5,77	28,80	15,60	6,39	38,10
First improvement ≤ 24 hours	2.87	1.57	5.23	3.32	1.72	6.40
First improvement ≤ 3 days	2.86	1.66	4.90	2.66	1.48	4.77
Response on Day 7	3.35	1.95	5.77	3.41	1.88	6.18
Response on Day 14	2.65	1.22	5.75	2.42	1.07	5.48
Recovery on day 7	1.16	0.69	1.95	1.12	0.62	2.00
Recovery on day 14	1.50	0.90	2.50	1.46	0.83	2.55
Very satisfied with treatment*	4.27	2.56	7.14	4.09	2.38	7.00
Choosing this therapy again*	12.26	5.07	29.66	14.72	5.65	38.40

Main outcomes: unadjusted odds ratios (Anthroposophy group vs. Conventional group) with 95% confidence intervals; odds ratios after multiple logistic regression analysis with adjustment for adjusting for gender, age, chief complaint, duration of complaint, complaint episode within last 12 months, baseline symptom score, concomitant disease present at baseline, previous treatment by the study physician. Odds ratio > 1 indicates better outcome in Anthroposophy group. Patients with data available for previous treatment by the study physician, n = 437. *At all available follow-ups.

Supplementary Table 7 Sensitivity analysis [f]: Odds ratios for main outcomes, adjustment also for body mass index

Outcome	Unadjusted			Adjusted		
	Odds ratio	95% confidence interval		Odds ratio	95% confidence interval	
		Lower margin	Upper margin		Lower margin	Upper margin
No antibiotics Days 0-28	6.24	2.72	14.30	6.04	2.35	15.52
No analgesics Days 0-28	9.59	3.54	25.96	15.78	4.82	51.69
First improvement ≤ 24 hours	2.80	1.43	5.49	3.28	1.55	6.94
First improvement ≤ 3 days	2.83	1.53	5.23	2.54	1.30	4.98
Response on Day 7	2.85	1.50	5.45	3.08	1.50	6.32
Response on Day 14	2.24	0.89	5.67	1.88	0.69	5.12
Recovery on day 7	1.17	0.64	2.15	1.13	0.58	2.23
Recovery on day 14	1.62	0.90	2.89	1.47	0.77	2.81
Very satisfied with treatment*	5.06	2.80	9.15	5.13	2.72	9.66
Choosing this therapy again*	11.23	3.97	31.78	12.81	3.88	42.25

Main outcomes: unadjusted odds ratios (Anthroposophy group vs. Conventional group) with 95% confidence intervals; odds ratios after multiple logistic regression analysis with adjustment for adjusting for gender, age, chief complaint, duration of complaint, complaint episode within last 12 months, baseline symptom score, concomitant disease present at baseline, body mass index. Odds ratio > 1 indicates better outcome in Anthroposophy group. Patients with data available for body mass index, n = 357. *At all available follow-ups.

Supplementary Table 8 Sensitivity analysis [g]: Odds ratios for main outcomes, adjustment also for household size

Outcome	Unadjusted			Adjusted		
	Odds ratio	95% confidence interval		Odds ratio	95% confidence interval	
		Lower margin	Upper margin		Lower margin	Upper margin
No antibiotics Days 0-28	6.75	3.36	13.54	6.88	3.20	14.79
No analgesics Days 0-28	13.29	5.94	29.72	16.20	6.49	40.45
First improvement ≤ 24 hours	2.70	1.48	4.94	3.11	1.61	6.04
First improvement ≤ 3 days	2.79	1.62	4.82	2.47	1.37	4.46
Response on Day 7	3.36	1.94	5.82	3.35	1.83	6.15
Response on Day 14	3.02	1.37	6.64	2.91	1.25	6.76
Recovery on day 7	1.18	0.69	2.00	1.21	0.67	2.18
Recovery on day 14	1.53	0.91	2.57	1.60	0.90	2.83
Very satisfied with treatment*	4.36	2.59	7.34	4.42	2.54	7.68
Choosing this therapy again*	12.61	5.21	30.53	15.03	5.72	39.53

Main outcomes: unadjusted odds ratios (Anthroposophy group vs. Conventional group) with 95% confidence intervals; odds ratios after multiple logistic regression analysis with adjustment for adjusting for gender, age, chief complaint, duration of complaint, complaint episode within last 12 months, baseline symptom score, concomitant disease present at baseline, household size. Odds ratio > 1 indicates better outcome in Anthroposophy group. Patients with data available for household size, n = 432. *At all available follow-ups.

Supplementary Table 9 Sensitivity analysis [h]: Odds ratios for main outcomes, adjustment also for household income

Outcome	Unadjusted			Adjusted		
	Odds ratio	95% confidence interval		Odds ratio	95% confidence interval	
		Lower margin	Upper margin		Lower margin	Upper margin
No antibiotics Days 0-28	8.24	3.31	20.50	8.18	2.92	22.90
No analgesics Days 0-28	14.86	5.44	40.60	15.36	4.93	47.86
First improvement ≤ 24 hours	3.35	1.42	7.94	4.96	1.90	12.94
First improvement ≤ 3 days	2.88	1.36	6.08	2.63	1.17	5.89
Response on Day 7	3.45	1.62	7.38	4.83	1.97	11.85
Response on Day 14	2.29	0.77	6.82	2.95	0.89	9.75
Recovery on day 7	1.07	0.52	2.21	0.95	0.42	2.14
Recovery on day 14	1.29	0.62	2.68	1.36	0.62	3.00
Very satisfied with treatment*	4.23	2.08	8.62	3.99	1.88	8.47
Choosing this therapy again*	11.67	4.18	32.58	12.18	3.94	37.63

Main outcomes: unadjusted odds ratios (Anthroposophy group vs. Conventional group) with 95% confidence intervals; odds ratios after multiple logistic regression analysis with adjustment for adjusting for gender, age, chief complaint, duration of complaint, complaint episode within last 12 months, baseline symptom score, concomitant disease present at baseline, household income. Odds ratio > 1 indicates better outcome in Anthroposophy group. Patients with data available for household income, n = 253. *At all available follow-ups.

Supplementary Table 10 Sensitivity analyses [i-k]: Odds ratios for main outcomes, adjustment also for caregiver's confidence in physician's professional skill and consultation length

Outcome	Unadjusted			Adjusted for variables											
				1-7: Main analysis			1-8: Sensitivity analysis [i]			1-7,9: Sensitivity analysis [j]			1-9: Sensitivity analysis [k]		
	OR	95% CI		OR	95% CI		OR	95% CI		OR	95% CI		OR	95% CI	
		LM	UM		LM	UM		LM	UM		LM	UM		LM	UM
No antibiotics Days 0-28	7.15	3.58	14.25	7.34	3.46	15.59	7.93	3.55	17.74	9.83	3.97	24.37	10.26	4.02	26.15
No analgesics Days 0-28	13.16	5.89	29.41	16.24	6.60	39.97	18.35	6.94	48.50	23.08	7.71	69.11	25.18	8.03	78.94
First improvement ≤ 24 hours	2.71	1.48	4.95	3.13	1.62	6.05	2.55	1.29	5.07	2.71	1.35	5.47	2.74	1.35	5.53
First improvement ≤ 3 days	2.76	1.60	4.75	2.57	1.44	4.58	2.02	1.10	3.72	2.78	1.47	5.25	2.83	1.49	5.38
Response on Day 7	3.50	2.03	6.04	3.48	1.91	6.32	2.82	1.52	5.25	4.04	2.08	7.85	4.11	2.11	8.01
Response on Day 14	3.04	1.38	6.68	2.74	1.20	6.30	2.03	0.84	4.87	2.06	0.83	5.07	2.18	0.88	5.44
Recovery on day 7	1.26	0.74	2.15	1.18	0.66	2.11	1.04	0.57	1.92	1.06	0.56	1.99	1.07	0.57	2.02
Recovery on day 14	1.63	0.97	2.73	1.58	0.90	2.77	1.40	0.78	2.52	1.38	0.75	2.53	1.38	0.75	2.54
Very satisfied with treatment*	4.10	2.45	6.87	3.94	2.30	6.75	2.54	1.40	4.61	3.06	1.72	5.44	2.26	1.19	4.26
Choosing this therapy again*	12.68	5.24	30.70	15.11	5.77	39.57	11.35	4.22	30.57	10.72	3.79	30.30	8.58	2.99	24.65

Main outcomes: unadjusted odds ratios (OR, Anthroposophy group vs. Conventional group) with 95% confidence intervals (CI), and odds ratios after multiple logistic regression analysis, adjusting for 1) gender, 2) age, 3) chief complaint, 4) duration of complaint, 5) complaint episode within last 12 months, 6) baseline symptom score, 7) concomitant disease present at baseline, 8) caregiver's confidence in physician's professional skill, 9) consultation length. Odds ratio > 1 indicates better outcome in Anthroposophy group. Patients with available data for all variables mentioned, n = 438. *At all available follow-ups. LM: Lower margin. UM: Upper margin.

Supplementary Figure 1 Patient recruitment and follow-up telephone interviews.

All evaluable patients had at least one interview.

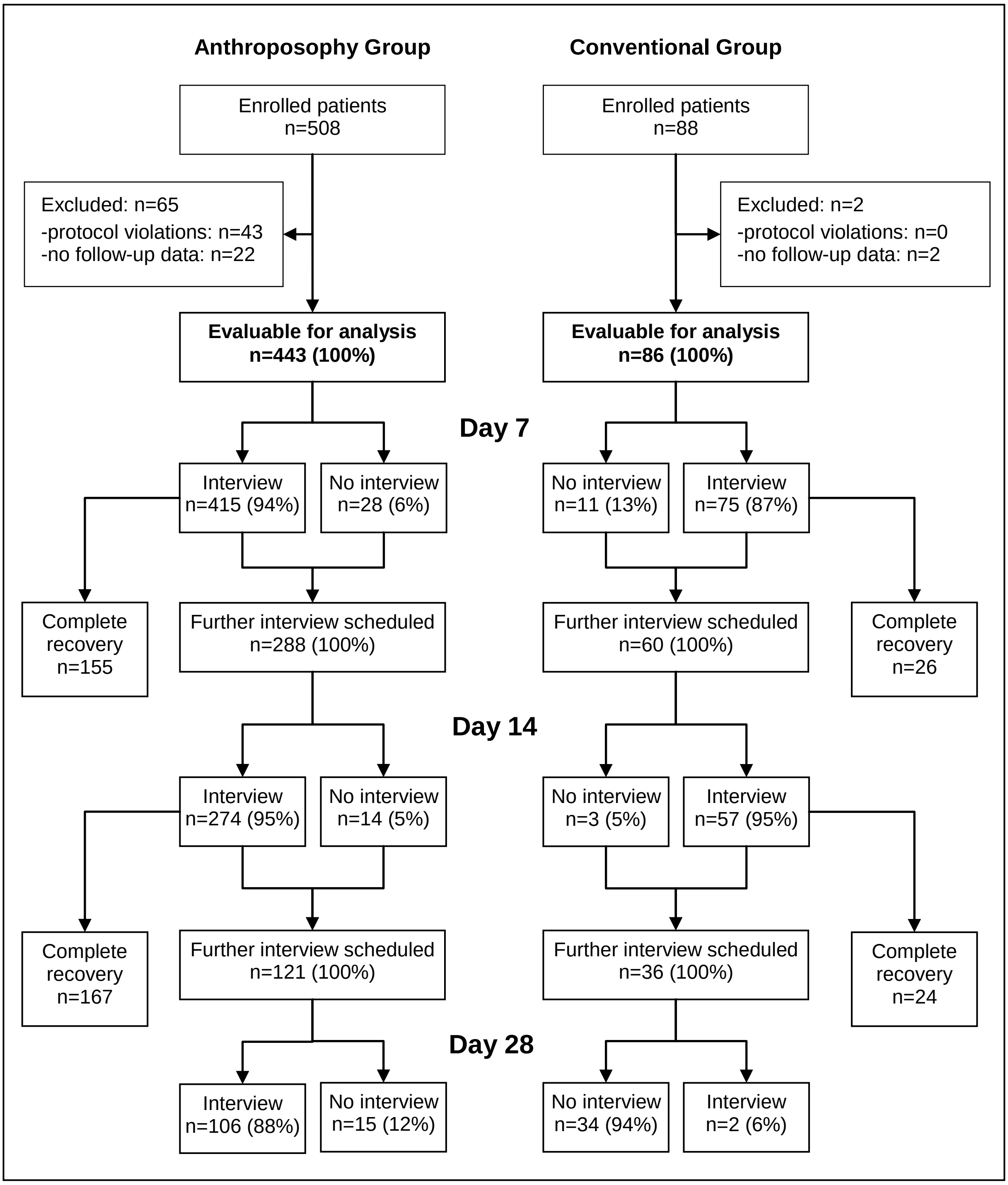

Supplement: Supplementary file 1 — Supplementary Table 1: Prescription of six most common Anatomical Therapeutic Chemical drug groups. Supplementary Table 2: Treatment outcome on Days 7, 14 and 28. Supplementary Table 3: Subgroup analysis of main outcomes according to chief complaint and age. Supplementary Table 4: Sensitivity analysis [a]: Odds ratios for main outcomes after exclusion of patients from the USA. Supplementary Table 5: Sensitivity analyses [b-d]: Odds ratios for main outcomes after substitution of one independent variable for another. Supplementary Table 6: Sensitivity analysis [e]: Odds ratios for main outcomes, adjustment also for previous treatment by the study physician. Supplementary Table 7: Sensitivity analysis [f]: Odds ratios for main outcomes, adjustment also for body mass index. Supplementary Table 8: Sensitivity analysis [g]: Odds ratios for main outcomes, adjustment also for household size. Supplementary Table 9: Sensitivity analysis [h]: Odds ratios for main outcomes, adjustment also for household income. Supplementary Table 10: Sensitivity analyses [i-k]: Odds ratios for main outcomes, adjustment also for caregiver's confidence in physician's professional skill and consultation length. Supplementary Figure 1: Patient recruitment and follow-up telephone interviews. [file 243801.f1.pdf]
